# Influenza A virus-dependent remodeling of pulmonary clock function in a mouse model of COPD

**DOI:** 10.1038/srep09927

**Published:** 2015-04-29

**Authors:** Isaac K. Sundar, Tanveer Ahmad, Hongwei Yao, Jae-woong Hwang, Janice Gerloff, B. Paige Lawrence, Michael T. Sellix, Irfan Rahman

**Affiliations:** 1 Department of Environmental Medicine Lung Biology and Disease Program, University of Rochester Medical Center, Rochester, NY, USA; 2 Department of Medicine, Division of Endocrinology, Diabetes and Metabolism, University of Rochester Medical Center, Rochester, NY, USA

## Abstract

Daily oscillations of pulmonary function depend on the rhythmic activity of the circadian timing system. Environmental tobacco/cigarette smoke (CS) disrupts circadian clock leading to enhanced inflammatory responses. Infection with influenza A virus (IAV) increases hospitalization rates and death in susceptible individuals, including patients with Chronic Obstructive Pulmonary Disease (COPD). We hypothesized that molecular clock disruption is enhanced by IAV infection, altering cellular and lung function, leading to severity in airway disease phenotypes. C57BL/6J mice exposed to chronic CS, BMAL1 knockout (KO) mice and wild-type littermates were infected with IAV. Following infection, we measured diurnal rhythms of clock gene expression in the lung, locomotor activity, pulmonary function, inflammatory, pro-fibrotic and emphysematous responses. Chronic CS exposure combined with IAV infection altered the timing of clock gene expression and reduced locomotor activity in parallel with increased lung inflammation, disrupted rhythms of pulmonary function, and emphysema. BMAL1 KO mice infected with IAV showed pronounced detriments in behavior and survival, and increased lung inflammatory and pro-fibrotic responses. This suggests that remodeling of lung clock function following IAV infection alters clock-dependent gene expression and normal rhythms of lung function, enhanced emphysematous and injurious responses. This may have implications for the pathobiology of respiratory virus-induced airway disease severity and exacerbations.

Influenza virus, rhinovirus, coronavirus, respiratory syncytial virus, parainfluenza, adenovirus and metapeumovirus are among the respiratory viruses known to cause exacerbations in Chronic Obstructive Pulmonary Disease (COPD) and asthma^1^,^2^,^3^,^4^. In fact, respiratory viral infections account for about 50–70% of acute exacerbations in patients with COPD^5^. COPD exacerbations are often followed by subsequent clinical pulmonary deterioration, including significant declines in forced expiratory volume at 1 second (FEV_1_) and increased hospitalization or mortality^6^. This is associated with worsening of COPD phenotypes, such as cough, fever and mucus production; the timing of which depends on the activity of the biological clock.

Circadian rhythms are biological oscillations that occur with a near-24-h period and are synchronized or entrained to environmental cues, such as the day-night cycle^7^. These rhythms are the manifestation of an autoregulatory molecular oscillator of interlocked positive and negative transcription factors collectively referred to as clock genes^8^. In mammals, the central pacemaker, localized to the suprachiasmatic nucleus (SCN) of the anterior hypothalamus, drives rhythms of physiology and behavior, and synchronizes internal timing with the external environment^7^. Apart from the central clock, peripheral tissues such as the liver, heart and the lung also contain autonomous circadian oscillators that coordinate tissue specific cellular functions and responses to environmental stimuli^7^,^8^,^9^,^10^,^11^,^12^. As an example, circadian rhythms in pulmonary function have been demonstrated in animal models and healthy individuals (highest at noon time and low during the early morning hours)^13^,^14^,^15^. The coordinated and synchronized activity of central and peripheral oscillators is referred to as the circadian timing system^9^,^12^. It has been shown that recruitment of leukocytes to tissues following infection is also regulated by the circadian timing system^16^,^17^. For example, macrophages and mast cells exhibit robust rhythms of circadian clock and proinflammatory cytokine gene expression^18^,^19^. The magnitude of immune and inflammatory parameters varies with time of day, and disruption of circadian rhythms (chronodisruption) has been implicated in cellular dysfunction, and in the pathogenesis of chronic metabolic disease, infection, and inflammatory diseases^20^,^21^,^22^.

An early morning surge in lung function and pronounced troughs in forced vital capacity (FVC), FEV_1_ and peak expiratory flow (PEF) during the night are common in patients with COPD exacerbations, including chronic smokers^23^,^24^. This could be due to cigarette smoke (CS)-mediated alterations in circadian clock proteins, levels of steroid hormones, surfactants in the lungs, mucus retention/secretion accompanied by increased inflammatory responses and a decline in normal rhythms of lung function^15^,^25^,^26^,^27^. Disruption of pulmonary function rhythms during COPD exacerbations commonly results in emergency room visits at night or in early morning hours when lung function is low^15^,^25^,^26^. In addition to a decline in lung function, patients with COPD have other sleep-related abnormalities, such as insomnia, excessive daytime sleepiness and altered rhythms of airway caliber and resistance^28^. Lung function is also altered following viral infections, including influenza A virus (IAV^29^), and IAV is known to increase the intensity and duration of exacerbations in those patients with COPD. However, the effect of virus-induced COPD exacerbations on clock function in the lung and the role of the lung clock in the pathogenesis of COPD and associated exacerbations are unknown.

We hypothesize that IAV infection exacerbates the effects of chronic CS-induced COPD/emphysema. Moreover, we hypothesize the negative consequences of IAV infection in chronic smokers and patients with COPD may be due to the combined influence of CS and influenza infection on circadian clock function in the lungs. To address these hypotheses, we measured body weight, mortality, locomotor activity, rhythms of clock and clock-controlled gene (CCG) expression in the lungs, lung function rhythms, inflammation and emphysematous responses of mice exposed to chronic CS with or without subsequent IAV infection. Further, to more directly assess the role of the timing system in response to infection, we measured activity, body weight, mortality, inflammation and emphysematous responses in the lungs of BMAL1 knockout mice and WT littermates following IAV infection.

## Results

### The negative effects of Influenza A virus infection on body weight, mortality and locomotor activity are exacerbated following chronic CS exposure

**Figure 1:**
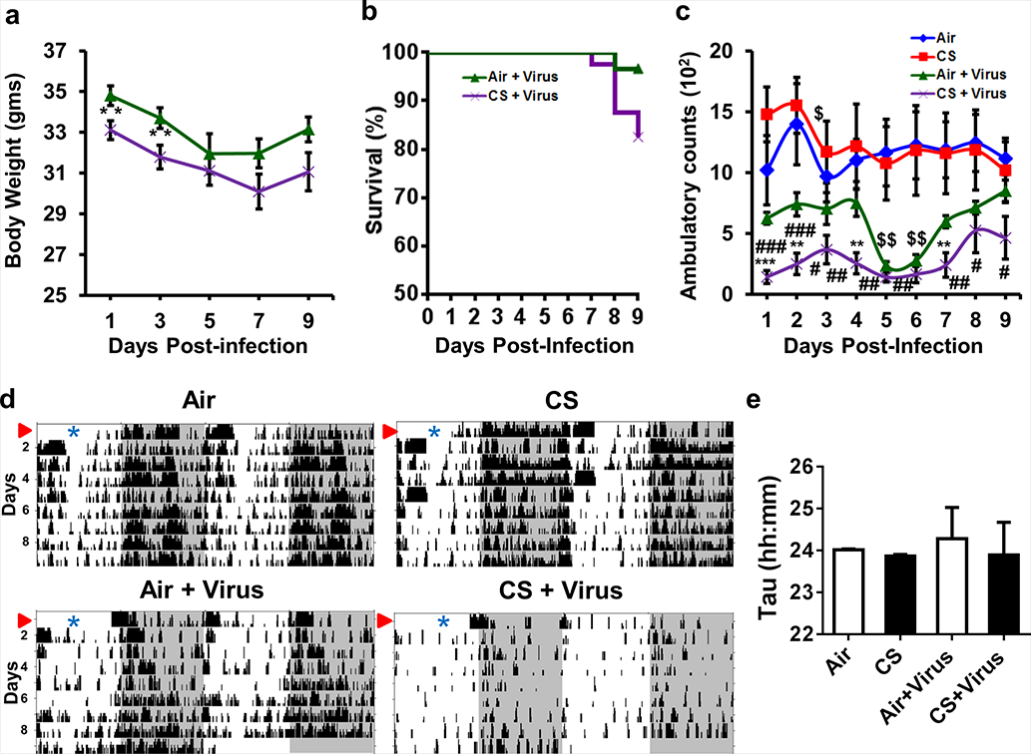
Influenza A virus infection differentially affects body weight, mortality and behavior in chronic air or CS-exposed mice. Data from uninfected air- and CS-exposed mice from a previous experiment were included for comparison [panels **c–e**;^30^]. **(a)** Chronic CS-exposed mice infected with IAV showed a modest reduction in weight, marked by a decline on days 1–3 post-infection relative to air-exposed mice infected with IAV. **(b)** Mortality was monitored for 9 days post IAV infection. Data are representative of mean ± SEM (n = 30–40 mice/group). ** *P* < 0.01 significant compared to Air+Virus group. Analysis of differences in survival over time was determined by a Mantel-Cox test (*P* < 0.68). **(c)** Nocturnal activity for 9 days post-infection was plotted as ambulatory counts. IAV infection reduced locomotor activity in CS-exposed mice by 70–80% during days 1–7 post-infection when compared to CS-exposed mice treated with saline. Similarly, IAV infection reduced locomotor activity in CS-exposed mice by 70–80% on days 1–2 and day 4 post-infection but only 30–45% during days 5–9 post-infection when compared to Air+Virus treated mice. Data are mean ± SEM (n = 6 mice/group) for each time point. ** *P* < 0.01; *** *P* < 0.001 significant compared to control groups (air or Air+Virus); ^$$ ^*P* < 0.01 significant compared to air-exposed mice; ^#^ *P* < 0.05; ^# #^ *P* < 0.01;^ # # # ^*P* < 0.001 significant compared to CS-exposed mice. **(d)** Representative double plotted actograms of total cage activity from chronic air, chronic CS, Air+Virus and CS+Virus treated mice. In panel **d**, gray shading indicates the dark phase (ZT12-24) and activity was not recorded during body weight measurements (ZT5-6). Red arrow head indicates day 0 IAV infection and asterisks denotes the time during which the mice were infected with IAV (ZT4-6). **(e)** Periodogram analysis of activity in L:D during days 1–8 post IAV infection. Period was very similar between air and CS groups, whereas the Air+Virus and CS+Virus groups showed increased variation in period though the mean was not significantly different from controls. Data are mean ± SEM (n = 6 mice/group).

Chronic (6 months) air- and CS-exposed mice were infected intranasally with 120 HAU IAV (H3N2). Before infection (Day 0), we did not observe a significant difference in body weight among the experimental groups. The body weight of mice in both IAV-infected groups dropped significantly on days 1–3 post-infection, with a slightly more dramatic decline in the CS+Virus group (Fig. 1a). This suggests that chronic CS-exposed mice exhibited greater mortality following IAV infection. Indeed, IAV infection in chronic CS-exposed mice produced a 14.1% reduction in survival within 9 days of infection (Fig. 1b). As reported, there was no significant difference in the body weight and mortality between the chronic air- and CS-exposed mice^30^. We found that chronic air and CS-exposed mice infected with IAV showed significant reduction in body weight compared to corresponding controls at the end of chronic exposure or day 9 post-infection (Supplementary Fig. 2b). Thus, while IAV infection alone (Air+Virus) has modest effects, infection of mice following chronic CS exposure (COPD/emphysema model) significantly increases mortality.

We have previously reported that mice exposed to 10 days of CS (acute exposure model) during the active phase show reduced locomotor activity associated with increased inflammation^30^. Similarly, mice exposed to chronic CS showed significantly reduced activity beginning 5 days after the first exposure that persisted for much of the exposure period^30^. In the present study, chronic CS-exposed mice infected with IAV (CS+Virus) showed a significant reduction in locomotor activity after infection that persisted for the duration of measurement (9 days post-infection; Fig. 1c–d). Chronic air-exposed mice infected with influenza (Air+Virus) showed an initial reduction in locomotor activity 1–5 days after infection with a trough 5–6 days after infection followed by a modest recovery by day 9 after infection (Fig. 1c–d). Actograms clearly reveal that the CS+Virus group showed significantly reduced locomotor activity post-infection (lower right panel) when compared to Air+Virus, air- and CS-exposed mice (Fig. 1d and Supplementary Fig. 2a). Limiting our analysis to the dark phase reveals that the primary influence of IAV infection was a reduction in nighttime activity, which was further suppressed in those mice previously exposed to chronic CS (Fig. 1c). The period of locomotor activity in L:D during days 1–8 post IAV infection was very similar between air and CS groups, whereas the Air+Virus and CS+Virus groups showed increased variation in period between animals although the mean was not statistically different from controls (Fig. 1e). Analysis of behavior as a function of daily distribution (day vs. night) emphasizes the effect of IAV treatment *in vivo* (Supplementary Fig. 3a-d). Control (air) and Air+Virus infected mice were primarily active (70–80% activity) during the dark phase and less active (20–30%) during the light phase from day 2 to day 9 post-saline infusion (Supplementary Fig. 3a and c). In three of the four groups, we detected a transient change in the distribution of activity on day 1 post-infusion regardless of treatment, suggesting that the infusion procedure acutely and temporarily altered activity (Supplementary Fig. 3a–d). In agreement with our previous report, chronic CS-exposed mice displayed a distribution of locomotor activity similar to air-exposed mice with the majority (70–80%) of activity limited to the dark phase (Supplementary Fig. 3b)^30^. Following IAV infection in chronic CS-exposed mice nighttime activity declined and daytime activity increased from days 3–7 post-infection such that we detected no difference between day and night activity levels by day 7 (Supplementary Fig. 3d). By day 8–9 post-infection the CS+Virus mice showed recovery of their normal nighttime (70–80%) and daytime (20–30%) distribution of activity (Supplementary Fig. 3d).

### The negative effects of Influenza A virus infection are enhanced in BMAL1 knockout mice

**Figure 2:**
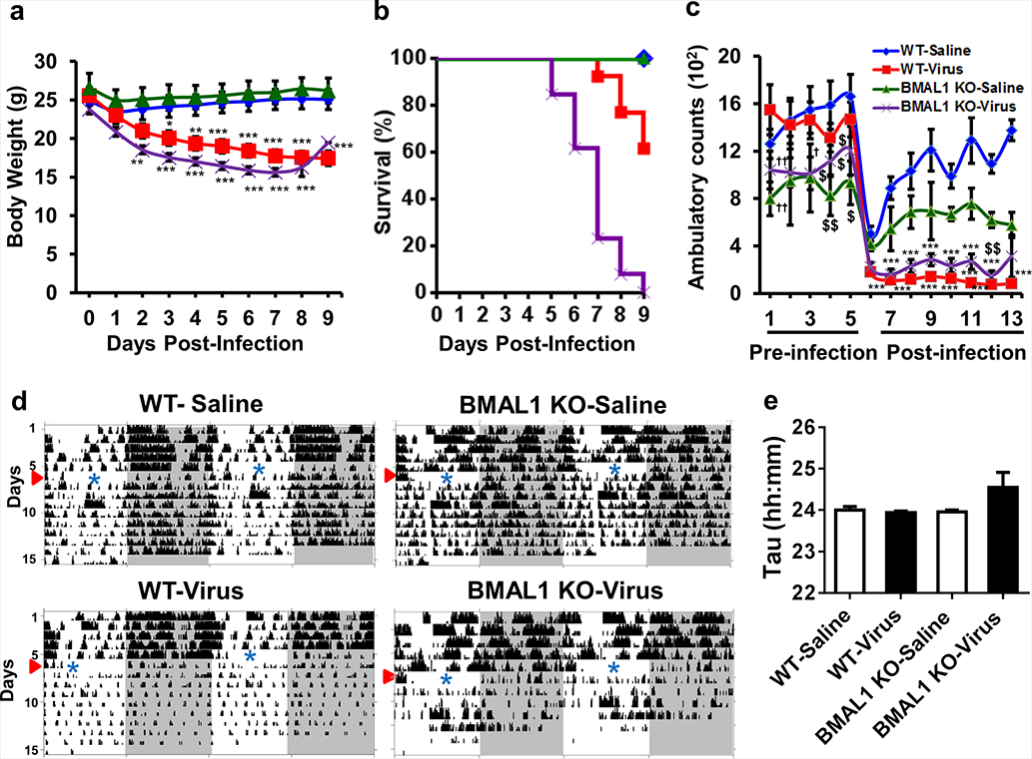
Influenza A virus infection differentially affects body weight, mortality and behavior in wild-type (WT) and BMAL1 KO mice. **(a)** IAV infection significantly reduced body weight of both WT and BMAL1 KO mice on day 2–9 post-infection compared to uninfected controls. * *P* < 0.05; ** *P* < 0.01; *** *P* < 0.001 significant compared to WT-Saline or BMAL1 KO-Saline; **(b)** Mortality was increased following IAV infection in both WT and BMAL1 KO mice, reaching 100% in BMAL1 KO mice within 9 days post-infection. Data are representative of mean ± SEM (n = 10 WT-Saline; n = 4 BMAL1 KO-Saline; n = 13 WT-Virus; n = 13 BMAL1 KO-Virus). Survival over time was analyzed with a Mantel-Cox test (*P* < 0.001). **(c)** Nocturnal activity of IAV infected mice (both WT and BMAL1 KO) was reduced on days 1–8 post-infection when compared to uninfected controls. ***P < 0.001 significant compared to uninfected controls; ^$ ^*P* < 0.05; ^$$ ^*P* < 0.01 significant compared to WT-Saline treated mice; ^††^ *P* < 0.01 significant compared to WT-Virus infected mice. **(d)** Representative double plotted actograms showing considerable reduction in total cage activity of IAV infected WT and BMAL1 KO mice relative to uninfected controls. In panel **d**, gray shading indicates the dark phase (ZT12-24) and activity was not recorded during body weight measurements (ZT5-6). Red arrow head indicates day 0 IAV infection and asterisks denotes the time during which the mice were infected with IAV (ZT4-6). **(e)** Periodogram analysis of activity in L:D was calculated during days 1–8 post-infection. Though there was no overall effect of IAV infection on period in either group an increase in variation was detected following infection in BMAL1 KO mice. Data are mean ± SEM (n = 10 WT-Saline; n = 13WT-Virus; n = 4 BMAL1 KO-Saline; n = 13 BMAL1 KO-Virus).

To dissect the role of clock regulatory proteins in response to respiratory infection, we measured the response of BMAL1 KO mice and wild-type littermates to IAV infection. Similar to mice that had been exposed to CS, BMAL1 KO and WT littermates were infected intranasally with 120 HAU (H3N2) IAV. Before IAV infection (Day 0), there was no significant difference in body weight between WT and BMAL1 KO mice (Fig. 2a). The body weight of WT and BMAL1 KO IAV infected mice dropped by day 2 post-infection and remained low until day 8 post-infection when compared to control mice of both strains (Fig. 2a). BMAL1 KO-Virus infected mice showed increased mortality beginning 5 days post-infection and by day 9 all of the BMAL1 KO mice infected with IAV was deceased (Fig. 2b). WT-Virus infected mice showed increased mortality beginning 7 days post-infection and reached a total of 38.5% mortality by day 9 post-infection (Fig. 2b). There was a significant difference in the body weight and mortality of WT-Virus compared to BMAL1 KO-Virus infected mice. This suggests that BMAL1 plays an important role in the host response to IAV infection. Further, our data suggest that circadian disruption may enhance the severity of infection, resulting in increased morbidity and mortality.

Locomotor activity of BMAL1 KO and WT littermates was monitored before (5 days) and after IAV infection (1–9 days post-infection). Regardless of genotype, IAV infection significantly reduced activity (Fig. 2c–d and Supplementary Fig. 2b). Both BMAL1 KO and WT mice showed significant reductions in daily activity on days 1–9 post-infection (Fig. 2c–d and Supplementary Fig. 2b). The period of locomotor activity in L:D on days 1–8 post IAV infection was very similar between all four groups, though IAV infected BMAL1 KO mice had greater variability in period relative to the other treatment groups (Fig. 2e). Analysis of behavior as a function of daily distribution (day vs. night) emphasizes treatment and genotype specific effects (Supplementary Fig. 4a–d). As expected, WT-saline treated mice were mostly active during the dark phase (ZT12-24; 70–80% of total activity) while BMAL1 KO-saline treated mice appeared to be arrhythmic and displayed a more even distribution of activity, though levels were still slightly higher at night (Supplementary Fig. 4a–b). IAV infection altered the normal distribution of daily activity in both WT and BMAL1 KO mice, though the effects were clearly more dramatic in WT mice (Supplementary Fig. 4c-d). WT mice infected with IAV increased their daytime activity but reduced their nighttime activity, producing apparent arrhythmia marked by a near equal distribution of activity across the 24h day (50% light; 50% dark). This effect of IAV infection on daily activity was less pronounced in BMAL1 KO mice, due largely to the fact that these mice already displayed a more equal distribution of daily activity/arrhythmia. That said, within 2 days of infection mean activity was actually higher in BMAL1 KO mice during the light phase, suggesting a brief period of diurnal preference following IAV infection (Supplementary Fig. 4d). This effect was diminished by day 3–4 post-infection with mice becoming more active during the night 5–9 days post-infection in parallel with increased mortality. These data reveal that, though BMAL1 KO mice differ from WT mice in terms of activity distribution prior to infection (most likely due to entrainment deficits in KO mice) both strains show reduced nocturnal preference following IAV infection.

### Influenza A virus affects the phase and amplitude of circadian clock gene expression in the lungs

**Figure 3:**
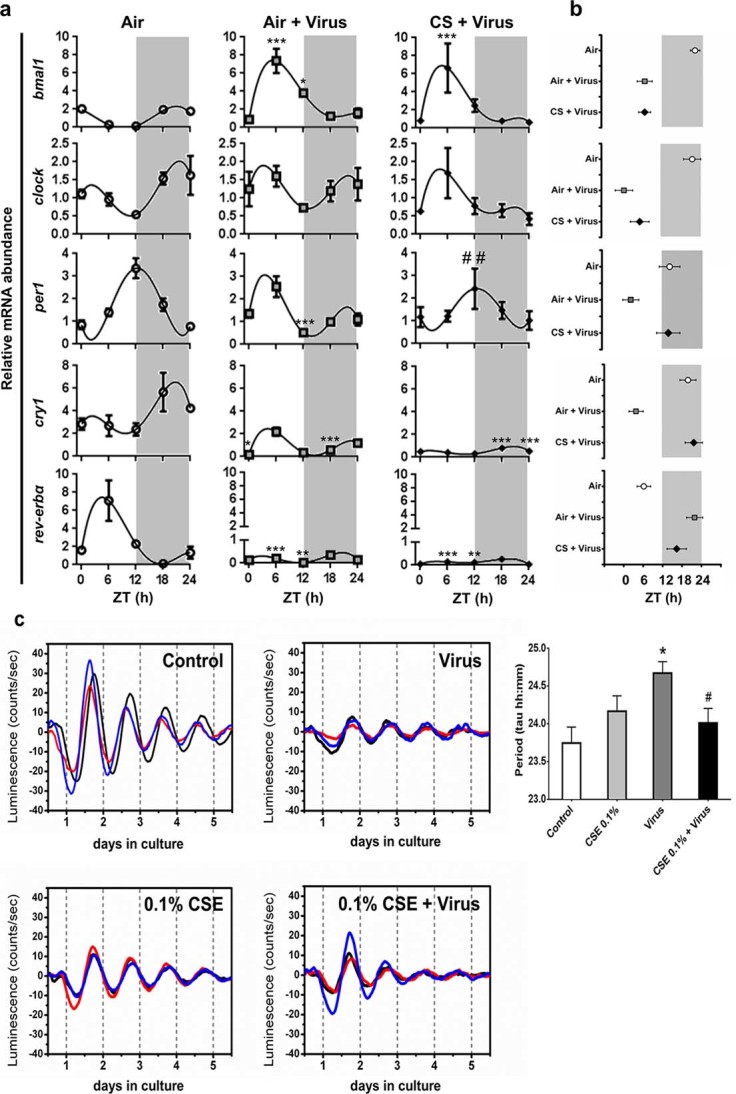
Diurnal rhythms of clock gene expression in the lungs are differentially affected by chronic CS and IAV infection. Data from uninfected air-exposed mice from a previous experiment were included for comparison [panels **a-b**;^30^]. Lung tissues were harvested every 6 h for 24 h beginning at ZT0 day 9 post-infection. **(a)** Expression of core clock genes (*bmal1, clock, per1, cry1,* and *rev-erbα*) in mouse lung tissue. CircWave analysis confirmed statistically significant rhythms of clock gene expression in Air+Virus (*P* < 0.05 for *per1* and *cry1*; *P* < 0.001 for *bmal1*) and CS+Virus (*P* < 0.05 for *per1*; *P* < 0.01 for *bmal1*) treated mice. **(b)** IAV infection adjusted the phase of clock gene expression in a gene- and treatment- (Air vs. CS) dependent manner**.** Center of gravity (COG) or peak phase for each clock gene was plotted on a horizontal phase map. In panels a and b, gray shading indicates the relative dark phase (ZT12-24). Data from air-exposed (open circle), Air+Virus (gray square) and CS+Virus (solid diamond) mice are representative of mean ± SEM (n = 3-4 mice/group) for each time point. * *P* < 0.05; ** *P* < 0.01; *** *P* < 0.001 significant compared to Air group. ^# #^ *P* < 0.01; significant compared to Air+Virus group. **(c)** Effect of CSE and IAV infection on the amplitude and period of PER2::LUC expression in lung tissue explants. PER2::LUC expression in representative lung tissue explants treated with medium alone (control), 0.1% CSE, virus alone (IAV, 300 HAU/ml), and 0.1% CSE+Virus. Treatment at the time of culture with 0.1% CSE and 0.1% CSE+Virus dampened the rhythm of PER2::LUC expression in lung tissue. Treatment with 0.1% CSE had a small effect whereas IAV infection significantly increased the period of PER2::LUC expression in lung explants. This effect was attenuated in explants treated with 0.1% CSE and IAV. Different colored traces represent tissue explants from different animals. Data are representative of mean ± SEM (n = 12-15/group). * *P* < 0.05 significant compared to control group; ^#^ *P* < 0.05 significant compared to Virus group.

We have previously reported that core clock genes (*bmal1, clock, per1-2, cry1-2, rev-erbα*) were rhythmically expressed in the lung of air- and CS-exposed mice both during acute and chronic CS exposure^30^. Of the known core clock genes, only *rorα* gene expression was not rhythmic in lungs of chronic air- and CS-exposed mice^30^. The peaks of gene expression rhythms for most clock genes in air-exposed controls were similar to those previously reported^30^,^31^. In both the chronic air- and CS-exposed mice, the expression of *bmal1, clock,* and *cry1* displayed nocturnal acrophases, peaking during the mid to late portion of the dark phase (ZT18-24; ZT0 = lights on: ZT12 = lights off)^30^. As anticipated, both chronic air- and CS-exposed mice showed peaks of *per1, per2, cry2, rev-erbα,* and *rev-erbβ* antiphase to *bmal1* (between ZT6-ZT12)^30^. Chronic CS-exposed mice showed a modest reduction in the amplitude of *bmal1* and *rev-erbα* expression and substantially reduced amplitude of *per1* expression^30^. Analysis of clock gene expression 9 days after IAV infection in air-exposed mice (Air+Virus) revealed significant rhythms of *bmal1* (*P* < 0.001), *per1, per2, cry1* and *rev-erbβ* (*P* < 0.05) but not *clock, cry2, rev-erbα* and *rorα* in lung tissue (Fig. 3a–b, Supplementary Fig. 5 and Table 1). Mice exposed to chronic CS and IAV infection (CS+Virus) only displayed rhythms of *bmal1* (*P* < 0.01) and *per1* (*P* < 0.05) expression in the lungs (Fig. 3a–b, Supplementary Fig. 5 and Table 1). Previously reported data from air-exposed mice are shown here for comparison (Fig. 3a–b, Supplementary Fig. 5 and Table 1). In Air+Virus infected mice, the peak expression of *bmal1, per1, per2, cry1, cry2,* and *rev-erbβ* shifted to the middle of the light phase (ZT6; ZT0 = lights on; ZT12 = lights off) and the amplitude of gene expression was altered at ZT6 when compared to air-exposed controls and chronic CS+Virus group (Fig. 3a–b, Supplementary Fig. 5a-b). As in Air+Virus treated mice, expression of *bmal1, clock* and *rev-erbβ* was shifted to peak at mid-day (ZT6) in CS+Virus exposed mice (Fig. 3b and Supplementary Fig. 5b). However, unlike Air+Virus treated mice, the phase of peak *per1* and *cry2* expression returned to ZT12 and the remaining clock genes [*per2, cry1, rev-erbα* and *rorα*] peaked at middle of dark phase (ZT18). Together, these data support the notion that IAV induces circadian disruption in the lungs, though the effects appear to be somewhat attenuated in mice exposed to chronic CS (Fig. 3a–b and Supplementary Fig. 5a–b).

We also determined the effects of CS with or without IAV infection on the expression of CCGs including *sirt1, ahr and muc5ac* in the lungs (Supplementary Fig. 5c–d). We have previously detected a circadian rhythm of *sirt1* expression that was altered by acute exposure to CS^30^. Herein we observed daily fluctuation of all three CCGs but only *ahr* rhythmicity was confirmed by CircWave analysis (*P* < 0.05). Though not statistically rhythmic in the air-exposed lungs, CircWave analysis did confirm significant rhythms of *sirt1* (*P* < 0.05) in the Air+Virus group that was attenuated by previous exposure to CS (Supplementary Fig. 5c-d and Table 1). Further, we detected a shift in peak *sirt1* and *ahr* expression from the latter portion of the dark phase (ZT18) in the air-exposed group to mid-day (ZT6) in both the Air+Virus and CS+Virus groups (Supplementary Fig. 5d). In addition to these phase shifts, there was an apparent increase in the amplitude of *sirt1* expression following IAV infection (Supplementary Fig. 5c). According to CircWave analyses *muc5ac* was not rhythmically expressed in lung tissue regardless of treatment (Supplementary Fig. 5c).

The effect of acute CS exposure combined with or without IAV infection on clock gene expression in the lung was confirmed by real-time monitoring of PER2::LUC expression in lung tissue explant cultures. Exposure to 0.1% cigarette smoke extract (CSE) *in vitro* produced a modest reduction in the amplitude and slight increase in the period of PER2::LUC expression in lung tissue explants in agreement with our previous report^30^. Viral infection (300 HAU/ml) also significantly reduced the amplitude and increased the period of PER2::LUC expression in lung explants (Fig. 3c). We observed comparable effects of 0.1% CSE+Virus similar to 0.1% CSE treatment alone in the amplitude and period of PER2::LUC expression in lung tissue explants (Fig. 3c). There was a significant reduction in the period of PER2::LUC expression in lung tissue explants treated with 0.1% CSE+Virus compared to IAV infection alone (Fig. 3c). Overall, CSE+Virus and IAV infection alone both reduced the amplitude and differentially affected the period of PER2::LUC expression in lung tissue explants.

### Infection with IAV increases macrophages, lymphocytes and CS-mediated inflammatory responses in the lungs

**Figure 4:**
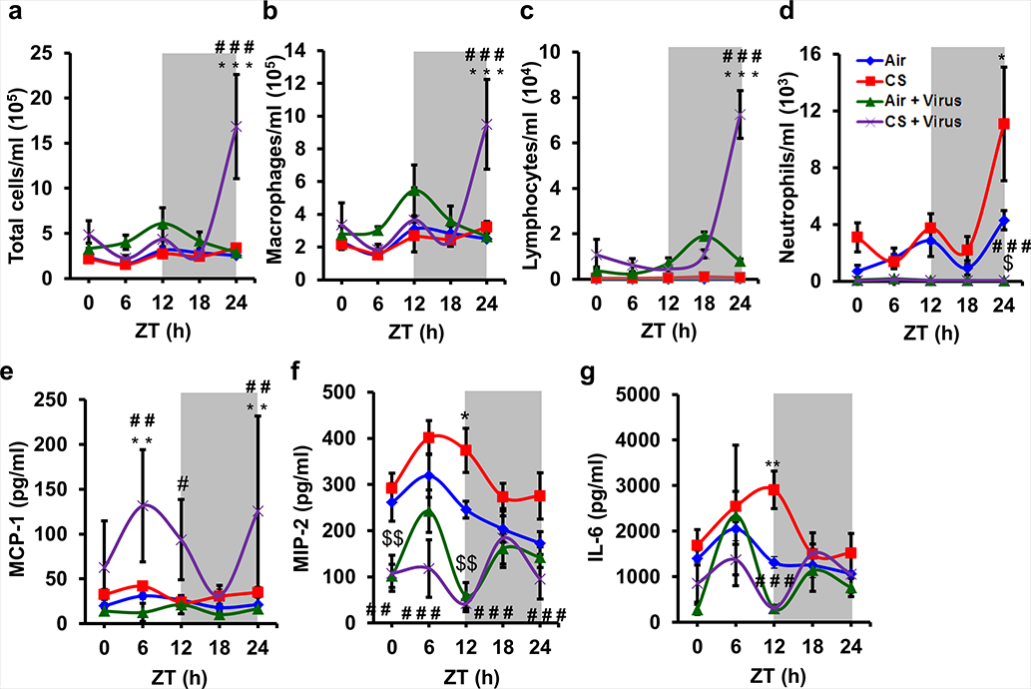
Chronic CS-exposed mice infected with influenza A virus show increased inflammatory cell influx and proinflammatory cytokine release in BAL fluid. Data from chronic (6 months) air- or CS-exposed mice given intranasal inoculation of either saline (control group) or influenza A virus (IAV; treatment group) at ZT4-6 are shown. Data from uninfected air- and CS-exposed mice from a previous experiment were included for comparison [panels **a–d**;^30^]. The total number of inflammatory cells was determined in BAL fluid from air, CS, Air+Virus and CS+Virus infected mice on day 9 post-infection. At least 500 cells in the BAL fluid were counted to determine **(a)** total cells, **(b)** total macrophages, **(c)** total lymphocytes and **(d)** total neutrophils. Data are representative of mean ± SEM (n = 3-4 mice/group) for each time point. * *P* < 0.05, *** *P* < 0.001 significant compared to air-exposed mice; ^$^ *P* < 0.05 significant compared to air-exposed mice; ^# # #^ *P* < 0.001 significant compared to CS-exposed mice. Levels of proinflammatory mediators **(e)** MCP-1, **(f)** MIP-2 and **(g)** IL-6 levels were measured in BAL fluid obtained from air, CS, Air+Virus and CS+Virus infected mice. IAV infection of chronic CS-exposed mice alters diurnal rhythms of proinflammatory cytokine release in mouse lungs. Data are representative of mean ± SEM (n = 3-4 mice/group) for each time point. * *P* < 0.05; ** *P* < 0.01; *** *P* < 0.001 significant compared to air or Air+Virus groups; ^#^ *P* < 0.05; ^# #^ *P* < 0.01; ^# # #^ *P* < 0.001 significant compared to CS-exposed mice.

As previously reported, similar levels of virus-specific antibodies were present in the blood of both Air+Virus and CS+Virus infected mice^32^. To investigate whether IAV infection after chronic CS exposure alters the inflammatory status in mouse lung, we measured the number of leukocytes in lung bronchoalveolar lavage (BAL) fluid 9 days post-infection. We observed more total cells, macrophages and lymphocytes near the end of the dark phase (ZT24) in the CS+Virus group when compared to Air+Virus, air- and CS-exposed mice (Fig. 4a–c). There was also a significant increase in neutrophils at ZT24 in chronic CS-exposed mice compared to air-exposed control. That is, in the absence of infection, there was an increase in neutrophils; however, this was not observed on day 9 of infection (Fig. 4d).

We next determined the impact of chronic CS exposure followed by IAV infection on rhythms of proinflammatory cytokine release in BAL fluid. MCP-1, IL-6 and MIP-2 levels were measured in BAL fluid at day 9 post-infection across the 24h day. The CS+Virus group showed significant increases in the expression of MCP-1 at ZT6 and ZT24 when compared to Air+Virus and air-exposed controls (Fig. 4e). We observed a significant decline in the levels of proinflammatory cytokines MIP-2 at ZT0 and ZT12 and IL-6 at ZT12 in the Air+Virus and CS+Virus groups 9 days post-infection compared to uninfected CS-exposed mice (Fig. 4f–g). Chronic CS-exposed mice showed significant increases in the levels of IL-6 and MIP-2 that peaked at ZT12 compared to air-exposed controls (Fig. 4f–g). Overall, IAV infection dampened the total number of neutrophils, MIP-2 and IL-6 levels, while it increased the total number of macrophages, lymphocytes and MCP-1 levels by day 9 post-infection. CircWave analysis confirmed significant diurnal rhythms of both IL-6 and MIP-2 levels in BAL fluid from chronic CS-exposed mice (*P* < 0.01). In contrast, chronic air-exposed, Air+Virus and CS+Virus groups did not show rhythmic expression of MCP-1, IL-6 and MIP-2 in BAL fluid as confirmed by CircWave analysis. The increase in levels of cytokines that we observed on day 9 post-infection was associated with influx of inflammatory cells in the lung in CS+Virus and chronic CS-exposed mice (Fig. 4a–g and Supplementary Fig. 6a-d).

We have also analyzed proinflammatory cytokine release in BAL fluid by pooling time points according to photoperiod (ZT6+ZT12 = daytime and ZT18+24 = nighttime) for MCP-1, MIP-2, IL-6 and TGF-β1. Chronic CS-exposed mice showed a significant increase in the levels of MIP-2, IL-6 at ZT6+ZT12 (daytime) and TGF-β1 at ZT18+ZT24 (nighttime) compared to controls (Supplementary Fig. 6a–d). Chronic CS-exposed mice infected with IAV showed a significant increase in the level of MCP-1 both at ZT6+ZT12 (daytime) and ZT18+ZT24 (nighttime). The levels of other cytokines including MIP-2, IL-6 and TGF-β1 were significantly reduced in CS+Virus group compared to CS-exposed mice at ZT6+ZT12 (Supplementary Fig. 6a-d). Thus, our data show that disruption of circadian clock function in the lung was associated with augmented MCP-1 levels during IAV-induced exacerbation in chronic CS-exposed mice.

To further examine the role of circadian regulatory factors on host responses to infection, we determined the total number of leukocytes in the airways. Consistent with prior reports, there was a significant increase in total cells, macrophages, lymphocytes and neutrophils in BAL fluid from WT-Virus infected mice compared to WT-Saline treated mice (Supplementary Fig. 7a–d). However, we were unable to evaluate immune cells in lungs of infected BMAL1 KO mice due to 100% mortality in this group by day-9 post-infection (see Fig. 2b). Because we observed a significant increase in inflammatory cellular influx into the lung of IAV infected WT mice, we next determined the level of proinflammatory cytokines/chemokines in BAL fluid. WT-Virus infected mice showed a significant increase in release of proinflammatory mediators such as IL-6, MCP-1, IP-10, KC, IL-10, and TNFα when compared to WT-Saline treated mice (Supplementary Fig. 8). The change in the lung proinflammatory mediators in BAL fluid observed at day 9 post-infection was correlated with influx of inflammatory cells in the lung (Supplementary Fig. 7a-d and Supplementary Fig. 8). Thus, our data show correlation of alteration in inflammatory cells in the lung with augmented pro-inflammatory cytokine responses during IAV infection *in vivo*.

### Influenza A virus augments chronic cigarette smoke-mediated inflammation and fibrosis

**Figure 5:**
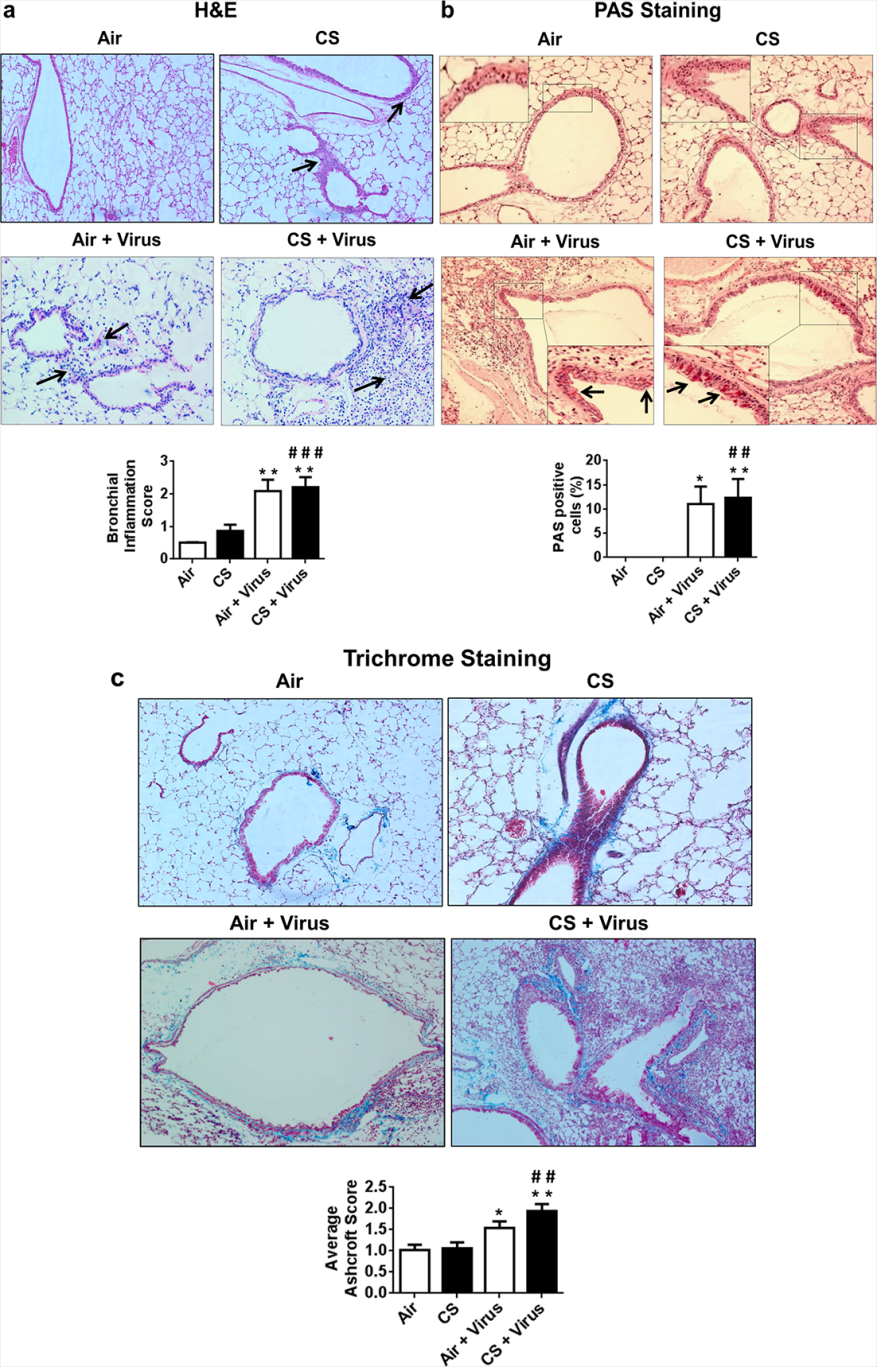
Chronic CS-exposed mice infected with IAV show persistent inflammation, mucus hypersecretion, and pulmonary fibrosis. Data from chronic (6 months) air- or CS-exposed mice given intranasal inoculation of either saline (control group) or influenza A virus (IAV; treatment group) at ZT4-6 are shown. Lungs were harvested on day 9 post-infection. **(a)** Representative images of lung tissues stained with hematoxylin and eosin (H&E) to demonstrate parenchymal and bronchial airway inflammation. Bronchial inflammation scores were calculated for each treatment group. **(b)** Representative images of lung tissues stained with Periodic-acid Schiff (PAS) to visualize mucus overproduction induced by IAV in the bronchial epithelium of chronic air- and CS-exposed mice. Average mucus scores from 3–4 different areas per slide/treatment group (n = 4-5mice/group) was used to calculate the percentage of PAS positive cells. **(c)** Representative images of lung tissues stained with Gomori’s Trichrome to visualize matrix accumulation/collagen deposition and quantified by Ashcroft fibrosis score. Original magnification x200. Data are representative of mean ± SEM (n = 4-5 mice/group). * *P* < 0.05; ** *P* < 0.01; significant compared to air- or CS-exposed mice. ^# #^ *P* < 0.01; ^# # #^ *P* < 0.001; significant compared to air-exposed mice.

Chronic CS exposure enhanced IAV induced inflammatory responses, as observed by increases in peribronchial and perivascular inflammatory cell infiltration in the airways (Fig. 5a). Mucus hypersecretion in the airways is an important characteristic pathological feature during acute exacerbation of COPD and is a key facet of inflammation-induced airway obstruction^33^. We determined the degree of mucus production in the airways of Air+Virus and CS+Virus infected mice at day 9 post-infection. Mucus-producing PAS positive cells were significantly increased in airway epithelium of chronic CS+Virus infected mice compared to Air+Virus infected mice that displayed a paucity of mucus producing cells in the airway epithelium (Fig. 5b). Trichrome staining demonstrated significant increases in airway fibrosis in mice exposed to CS+Virus with increase in collagen deposition observed around the airways (Fig. 5c). Further, we detected a significant increase in α-SMA levels, a marker for activated myofibroblasts observed in fibrotic lung disease, in the fibrotic regions of the lung from both air and CS-exposed mice infected with IAV (Supplementary Fig. 9a). These characteristic features suggest that IAV infection of mice previously exposed to chronic CS results in exaggerated lung inflammation and fibrotic airway remodeling *in vivo*. It has been documented that after IAV infection, epithelial damage induced by the virus could be repaired in order to completely restore lung structure and function *in vivo*. Clara cell specific marker, CCSP was used to identify changes that occur during virus infection in the airway bronchial epithelial cell population. At day 9 post-infection, we observed a significant decrease in the expression of CCSP positive cells in the airway bronchial epithelium of chronic CS+Virus infected mice compared to Air+Virus, air- and CS-exposed mice (Supplementary Fig. 9a).

### Influenza A virus exaggerates chronic cigarette smoke-mediated airspace enlargement/emphysema and altered rhythms of lung function

To investigate the role of IAV-induced circadian clock disruption of the lungs and the possible impact on lung airway remodeling and pulmonary function, we examined airspace enlargement/emphysema by lung histopathologic and functional measurements in chronic CS+Virus infected mice. There was a significant difference in lung histopathological changes between chronic CS+Virus infected mice and Air+Virus infected mice 9 days post-infection, which was confirmed with measurements of the mean linear intercept (MLI; Air+Virus 43.27 ± 3.31 vs. CS+Virus 62.27 ± 3.20; *P* < 0.001; Supplementary Fig. 9b). These data suggest that IAV infection and chronic CS exposure can synergistically enhance airway remodeling in mice.

**Figure 6:**
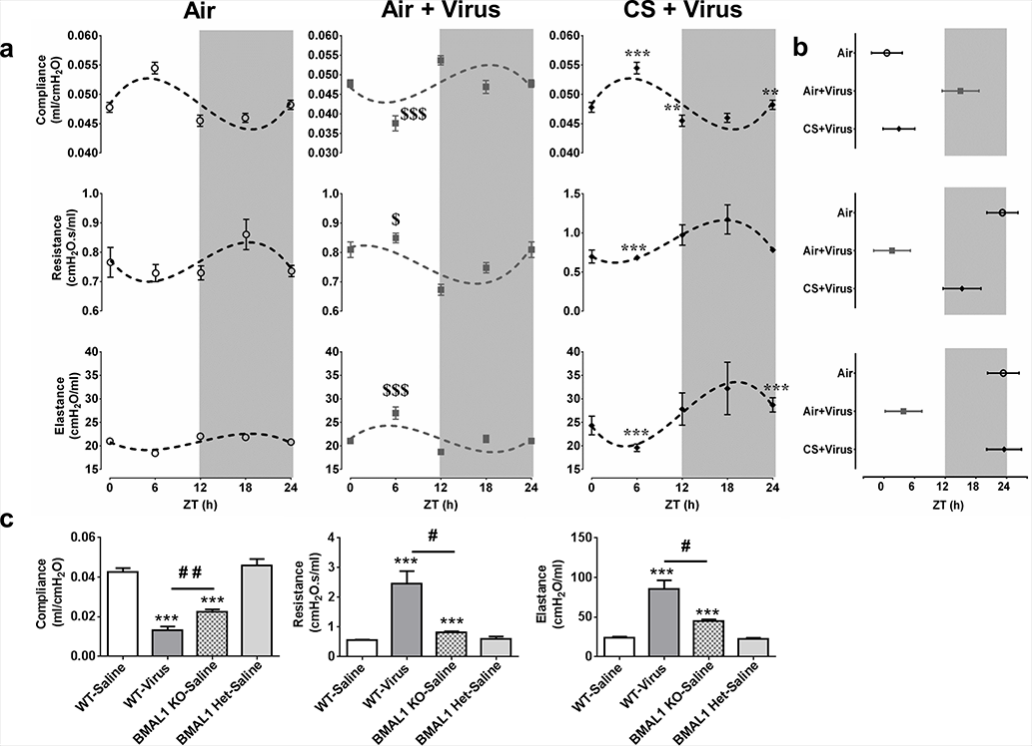
Daily rhythms of lung function are differentially affected by chronic CS-exposure and influenza A virus infection. Data from chronic (6 months) air- or CS-exposed WT mice given intranasal inoculation of either saline (control group) or influenza A virus (IAV; treatment group) at ZT4-6 are shown. Data from uninfected air-exposed mice from a previous experiment were included for comparison [panels **a–b**;^30^]. **(a)** Daily rhythms of compliance, resistance and elastance were measured in air, Air+Virus and CS+Virus mice. As in previous experiments, measurements were taken on day 9 post-infection. **(b)** COG or peak phase values for each measure of lung function were plotted on a horizontal phase map. Gray shading in panels a and b indicates the relative dark phase (ZT12-24). Data from air-exposed (open circle), Air+Virus (gray square) and CS+Virus (solid diamond) are representative of mean ± SEM (n = 3-4 mice/group) for each time point. ** *P* < 0.01; *** *P* < 0.001, significant compared to Air+Virus; ^$^ *P* < 0.05; ^$$$^ *P* < 0.001 significant compared to air-exposed mice. **(c)** Influenza A virus infection altered lung function in WT mice. After day 9 post-infection, lung compliance, resistance and tissue elastance were determined in WT-Saline, WT-Virus, BMAL1 KO-Saline and BMAL1 Het-Saline (heterozygous) treated mice. Data are representative of mean ± SEM (n = 10 WT-Saline; n = 8 WT-Virus, n = 4 BMAL1 KO-Saline and n = 6 BMAL1 Het-Saline) for each time point. *** *P* < 0.001, significant compared to WT-Saline; ^#^ *P* < 0.05; ^# #^ *P* < 0.01 significant compared to WT-Virus.

We then determined the impact of IAV infection on diurnal rhythms of lung function, including lung compliance, resistance and tissue elastance. We have previously reported that when examined as a function of time of day (day vs. night) chronic CS-exposed mice show significantly increased lung compliance and reduced elastance, but resistance is not significantly reduced^30^. When analyzed at specific time points across the day, elastance and resistance were only significantly reduced at ZT18 in chronic CS-exposed mice^30^. Lung compliance was significantly decreased at ZT12 and ZT24 in the CS+Virus group when compared to the Air+Virus group (Fig. 6a–b; Tables 2–3 and Supplementary Tables 1–3). There was a change in the phase and rhythms of lung compliance in Air+Virus group when compared to air-exposed and chronic CS+Virus infected mice (Fig. 6a–b; Tables 2–3 and Supplementary Tables 1–3). However, lung resistance decreased significantly only at ZT6 in CS+Virus group as compared to Air+Virus group (Fig. 6a–b; Tables 2–3 and Supplementary Tables 1–3). Tissue elastance was significantly decreased at ZT6 and significantly increased at ZT24 in the CS+Virus group when compared to the Air+Virus group (Fig. 6a–b; Tables 2–3 and Supplementary Tables 1–3). Similarly, there were significant alterations in the phase and amplitude of lung resistance and tissue elastance rhythms in CS+Virus group and Air+Virus group when compared to air-exposed controls (Fig. 6a–b; Table 2–3 and Supplementary Tables 1–3). Overall, when we combined the data from day (ZT6 and ZT24) and night (ZT12 and ZT18), lung compliance was significantly reduced whereas lung resistance and tissue elastance significantly increased during the dark phase (ZT12-18) in CS+Virus group compared to Air+Virus group (Fig. 6a–b; Tables 2–3 and Supplementary Tables 1–3). Thus, IAV infection appeared to invert the phase of peak tissue elastance and resistance but not lung compliance such that each marker of pulmonary function peaked during the light phase (when the animal is generally sleeping; Fig. 6b). Exposure to CS prior to IAV appeared to oppose this response, preventing the dramatic shifts produced by IAV infection.

### BMAL1 KO mice show altered lung function and pro-fibrotic responses

To investigate the role of the molecular clock in the response to IAV infection, we measured lung compliance, resistance and tissue elastance in WT mice infected with IAV on day 9 post-infection, uninfected BMAL1 heterozygous KO mice and uninfected homozygous BMAL1 KO mice. Lung compliance was significantly decreased in both WT-Virus and BMAL1 KO-Saline treated mice compared to WT-Saline and BMAL1 Het-Saline treated mice (Fig. 6c). However, lung resistance and tissue elastance were significantly increased in both WT-Virus and BMAL1 KO-Saline treated mice (Fig. 6c). BMAL1 KO-Saline treated mice displayed a significant increase in lung compliance and decrease in resistance and elastance when compared to WT-Virus infected mice suggesting an inherent defect in the lung mechanical properties of BMAL1 KO mice in the absence of infection (Fig. 6c). In a separate experiment, we determined lung function at different ZT (ZT0-ZT18; n = 1 mice/ ZT time point) in 2–3 months old BMAL1 KO mice and WT littermates. We also performed differential cell counts in BAL fluid collected from WT and BMAL1 KO mice at all the four ZT time points. We averaged the cell counts data from different time points and found that Bmal1 KO mice had a significant increase in neutrophil counts compared to WT littermates (Supplementary Fig. 10a). Total cell counts and macrophage counts were not significantly different between BMAL1 KO and WT littermates (Supplementary Fig. 10a).

We measured the rhythms of lung mechanical properties in BMAL1 KO and WT littermates at different ZT time points. BMAL1 KO mice showed similar peaks of lung function measurements as air-exposed controls but each was significantly altered (reduced compliance, increased resistance and elastance) compared to WT littermates (Supplementary Fig. 10b). We found that BMAL1 KO mice develop spontaneous pro-fibrotic lung phenotype as they age when compared to WT littermates (Supplementary Fig. 10c). The development of a fibrotic-like phenotype in BMAL1 KO mice could contribute to the altered lung function we observed in these mice (Fig. 6c). Thus, our data suggest that the clock gene activator BMAL1 may play an essential role in maintaining normal pulmonary function. Influenza A virus infection in BMAL1 KO mice further enhance the severity of virus infection resulting in increased morbidity and mortality.

## Discussion

Patients with COPD display daily rhythms of symptom exacerbation affiliated with sleep disruption^34^,^35^,^36^. Given the daily nature of these exacerbations and their impact on sleep quality, we hypothesized that disruption of the biological timing system occurs during the etiology of COPD. We have previously reported that rhythms of behavior (locomotor activity), hormone secretion (serotonin and corticosterone) and clock gene expression in the lungs and brain are altered in a mouse model of COPD due to environmental tobacco/cigarette smoke exposure^30^,^37^. Influenza infection is also known to alter behavior^38^,^39^ and has a negative influence on lung function^29^. The immune-inflammatory system is regulated by the circadian clock at multiple levels with immune and inflammatory responses showing robust daily oscillations^20^,^21^. Though limited evidence suggests that IAV infection alters sleep patterns via targeted effects on gene expression in particular hypothalamic sleep centers^40^, it has yet to be determined if IAV infection alters circadian clock function directly in the lungs or affects daily rhythms of lung function. Further, the potential for exacerbation of COPD impacts on clock function due to IAV infection has not been determined. Using a mouse model of COPD, we have determined the impact of IAV infection alone or IAV infection comorbid with COPD on clock function in the lungs, activity levels, inflammatory/injurious lung responses and daily rhythms of lung function. To address the role of the clock during COPD and/or infection, we carried out these experiments in *bmal1* knockout mice that lack a functioning oscillator. We demonstrate that IAV infection further exacerbates the effects of COPD on clock function in the lungs and daily rhythms of behavior. Further, our data reveal a potentially critical role for the circadian clock in mitigating a normal immune response to IAV infection.

Patients with COPD have poor sleep quality, increased sleep latency, decreased total sleep time, increased waking after sleep onset and decreased non-REM and REM sleep episodes^36^. These individuals are also commonly diagnosed with sleep disorders including obstructive sleep apnea^41^. Psychological distress in those with COPD is associated with a decline in lung function, increased exacerbation frequency and worsening of cardiovascular disease, further disrupting sleep in these patients^34^. Though our experiments did not directly measure sleep *per se* (e.g. using polysomnographic recording), the considerable reduction in activity during the day and night in both WT and BMAL1 KO mice following IAV infection implies adaptive changes in the homeostatic or circadian sleep drive that lead to increased inactivity during IAV infection. This increase in sleep/reduced activity level could be as a result of immune/inflammatory cell infiltration (e.g. macrophages) that can generate influenza-induced sleep enhancement^38^. Alternative compensatory immune mechanisms that generate an effective host defense response may also contribute to sleep propensity during viral infection^38^. It has been suggested that altered expression of *Temt* (thioether-S-methyltransferase) in the hypothalamus and basal forebrain during IAV infection may influence sleep patterns through its effects on prostaglandin metabolism^42^.

We show for the first time that IAV infection alters circadian clock gene expression in the lungs and reduces the amplitude of locomotor activity in a COPD/emphysema mouse model. The effect of IAV infection persisted longer (post-infection day 7–9) in chronic CS-exposed mice, coincident with a decrease in body weight and increased mortality. BMAL1 KO mice infected with IAV also displayed a significant decline in body weight and survival (100% mortality), suggesting that proper function of the timing system is necessary for maintaining the innate immune response to infection. Chronic air- and CS-exposed mice infected with IAV after 6 months of exposure and WT and BMAL1 KO mice infected with IAV show widely varying changes in morbidity (weight) and mortality. This could be due to strain background- and age-dependent effects of IAV infection. The mice were 2–3 months old when we started the chronic air and CS exposure. At the end of chronic air and CS exposure (6 months) and before IAV infection chronic air- and CS-exposed mice were about 9–10 months old. Similarly, WT littermates and BMAL1 KO mice were 2–4 months old when they were infected with IAV. Hence, we speculate the two confounding factors such as strain background and age of these mice when they were infected with IAV play an essential role on the severity of IAV on the loss of body weight and mortality *in vivo*. This notion is supported by studies wherein circadian desynchronization due to experimental jet-lag increased inflammatory responses and mortality following LPS challenge^43^. It is evident that changes in the phase and amplitude of clock gene expression in the lungs are affiliated with impaired lung function (compliance, resistance and tissue elastance). Analysis of lung function rhythms clearly revealed a near inversion of peak phase rhythms of lung function in Air+Virus group compared to air-exposed mice. Though generally similar, prior exposure to CS (COPD) did not fully attenuate the effects of IAV infection, as there were still slight differences in peak lung functions in these mice. These changes in lung function rhythms may underlie the reduced activity and increased mortality observed among IAV infected mice.

To assess the impact of IAV with or without CS exposure more directly, we examined the influence of cigarette smoke extract (CSE) and influenza A virus (300 HAU/ml) on PER2::LUC expression in lung tissue explants. As reported earlier, CSE treatment at a very low dose (0.1%) tended to lengthen the period of PER2::LUC expression in lung tissue explants, though the effect was modest. Infection with IAV resulted in a significant increase in period when compared to uninfected controls. Surprisingly, CSE treatment combined with IAV infection attenuated the effect of IAV infection alone on the period but not the amplitude of PER2::LUC expression in lung explants. Circadian disruption due to chronic jet lag has been shown to alter lung mechanics and clock gene expression in the lungs in a sexually dimorphic manner^44^. As a whole these data strongly suggest that CS exposure and virus infection either alone or in combination can affect clock function in the lungs. CS combined with IAV infection affects both lung CCGs and pulmonary function akin to other models of circadian disruption, suggesting that even a subtle change in clock function may have significant impact on clock-dependent physiological processes in both the lungs and immune system^16^,^20^,^21^,^45^,^46^. Though implied, direct support for a functional link between rhythms of lung function and circadian clock gene expression in the lungs remains elusive. While not conclusive evidence for such a link, the combined and somewhat parallel impacts of IAV infection on pulmonary function and clock gene expression in the lungs support the notion that the lung clock contributes in a meaningful way to the timing of pulmonary physiology.

It is well known that immune-inflammatory parameters change with time of day and disruption of circadian rhythms has been associated with infectious and inflammatory diseases^20^,^21^,^45^. Studies from animal models highlight the extent to which the core clock proteins (BMAL1, CLOCK and REV-ERBα) regulate fundamental aspects of the immune-inflammatory response^21^, such as toll-like receptor 9 (TLR9)^46^ and repressing chemokine (C-C motif) ligand 2 (CCL2) expression^47^. Further, REV-ERBα has been shown to attenuate the activation of IL-6 expression^19^,^47^. It has been shown that the core circadian clock protein was bound to nuclear factor kappa B (NF-κB) RelA/p65 activating NF-κB-dependent transcription^48^. Both transcription factors activator protein 1 (AP-1) and NF-κB share unique sequences that overlaps consensus sequence from *rev-erbα* promoters demonstrating role of REV-ERBα in regulating oxidative stress and/or inflammation^49^. These reports suggest involvement of these proinflammatory gene regulatory transcription factors and role of molecular clock on exaggerated inflammatory responses observed in our mouse model of COPD.

BMAL1 KO mice are behaviorally arrhythmic^50^ and show signs of advanced aging and underlying pathologies, correlated with increased levels of ROS and cellular senescence^51^,^52^. We have recently shown that CS exposure reduced mRNA and protein levels of BMAL1 and BMAL1-CC10 cre (epithelium specific Bmal1 KO) with augmented inflammatory responses and dysregulation of CS-induced oxidative stress. These data suggest the involvement of the molecular clock in regulation of CS-induced lung inflammation^30^, which supports previous work indicating that BMAL1 has an anti-inflammatory function^19^,^53^. Recently, Clock^Δ19^ circadian mutant mouse lung showed altered temporal Nrf2 activity complemented with reduced GSH levels, increased protein oxidation and a spontaneous fibrotic-like phenotype^54^. We found that BMAL1 KO mice also develop a pro-fibrotic phenotype in the lung which could be due to increased ROS levels and altered oxidative stress-mediated cellular senescence^51^,^52^. BMAL1 deletion in myeloid cells demonstrated diurnal variation in the absolute number of specific monocytes (Ly6C^hi^) in blood and in the spleen under normal conditions which was enhanced during inflamed peritoneum at ZT8 versus ZT0^53^. This study also revealed that BMAL1 binds to E-boxes in the promoters of *Ccl2*, *Ccl8* and *S100a8* (encoding S100 calcium binding protein A8) and recruits with it members of the polycomb repressor complex (PRC2) thereby allowing repressive histone marks to block transcription and attenuate Ly6C^hi^ monocyte numbers and inflammation at the site of damage^53^. It has been shown that the number of leukocytes in the mouse circulation strongly correlates with circadian variability, such that leukocyte numbers peak at ZT5 and recruitment into the tissues peaks at ZT13^17^. Keller *et al.* demonstrated that the role of entire toll-like receptor 4 (TLR4) pathway in peritoneal macrophages is tightly regulated under the control of circadian clock and thus equip the immune cell to face exaggerated response at times^18^. In the present study, analysis of clock gene expression revealed a significant impact of IAV infection on the expression of core clock genes. There was also a time of day-dependent increase in the number of total cells, macrophage counts and lymphocyte counts following IAV infection of CS-exposed mice. In the present study, the total numbers of inflammatory cells were significantly increased at ZT24 when compared to ZT0 though they represent the same circadian phase. Previous reports suggest that inflammatory cells in the lungs were significantly increased in BAL fluid of mice euthanized 24 h after the last exposure compared to 2 h post-last CS exposure^55^,^56^. These findings suggests that CS exposure has a suppressive effect on the number of inflammatory cells recovered in the lavage, possibly due to capillary trapping or increased adhesion, which causes reduction in the numbers recovered from the air spaces at ZT0 compared to ZT24. This finding is supported by increased proinflammatory mediators released into the lung at 24 h compared to 2 h after the last CS exposure, which would attract more inflammatory cells into the lung interstitium during CS exposure.

The proinflammatory cytokine MCP-1 response was also greater in the IAV infected mice exposed to CS. Similarly, chronic CS exposure also increased MIP-2 and IL-6 at ZT6/12 when compared to controls. However, IL-6 and MIP-2 levels were dampened in CS plus Virus group which could be due to immunosuppressive effects of CS which is in line with recent report in which CS exposure suppressed the production of cytokines and chemokines after pandemic H1N1 or avian H9N2 virus infection in mice^57^. It has been shown that the IAV-mediated inflammatory response begins early on day 3 and remains high until day 5–7 post-infection^58^. Subsequently, viral clearance occurs by day 10 in the lungs, thereby resolving the inflammatory phenotype, such as inflammatory cellular influx and proinflammatory mediators release observed during IAV infection *in vivo*^58^. Hence, we did not observe significant increases in pro-inflammatory mediators at day 9 post IAV infection in chronic air- and CS-exposed mice. CS-exposed mice infected with IAV showed sign of severe pulmonary inflammation, lung permeability damage and mucus hypersecretion which are characteristic features of acute exacerbation of COPD^59^,^60^. We have previously reported that mainstream CS exposure at a concentration of 300 mg/m^3^ (TPM) for 8 weeks causes significant increase in PAS positive cells in mouse lungs^61^. In this study, we used a low dose side-stream smoke exposure (~90–100 mg/m^3^) for 6 months, that unlike mainstream smoke, does not cause mucus production in the chronic CS-exposed mouse lungs when compared to Air+Virus and CS+Virus groups. A recent study identified a regulatory mechanism, whereby the lung epithelial clock and glucocorticoid hormones control both time-of-day variation and magnitude of pulmonary inflammatory responses to bacterial infection^62^. Similarly, rhythms of pulmonary function define time-of-day dependent sensitivity to steroids and β2-agonists in patients with nocturnal asthma and asthmatics who smoke^63^,^64^,^65^. Hence, it is possible that the mechanism that couples the circadian clock and bronchiolar glucocorticoid receptor to pulmonary innate immunity plays an essential role during COPD exacerbations by IAV infection. These data suggest that temporal increases in chemoattractants, leukocyte trafficking, proinflammatory cytokines/chemokines, and phagocytic ability before the activity phase is indicative of clock-controlled sensitivity and immunosurveillance. Collectively, these data suggest that the molecular clock and associated transcription factors, epigenetic regulators, and key regulatory signaling pathways play an essential role in cytokine gene expression through temporal gating of immune responses.

Several studies have examined the effects of acute CS exposure combined with IAV infection^58^,^66^,^67^,^68^,^69^. Only two prior reports determined the effects of IAV infection combined with chronic CS exposure model^70^,^71^. Robbins *et al.* were the first to show chronic CS affects primary antiviral immune-inflammatory responses, yet secondary immune protection remained intact suggesting exaggerated inflammatory responses during viral infection might possibly influence decline in clinical status associated with COPD exacerbations^70^. Wortham *et al.* demonstrated NKG2D stimulation during chronic CS exposure plays an essential role in the development of NK cell hyper-responsiveness and influenza-mediated exacerbations of COPD^71^. Based on the published studies, it is evident that both acute and chronic CS exposure combined with influenza infection causes increased pulmonary and systemic inflammation *in vivo* which was accompanied by increased viral proliferation or reduced clearance^58^,^67^. In this study, the dynamics of viral proliferation were not affected; instead we observed an exaggerated inflammatory response and apparent normalization of lung function rhythms in chronic CS-exposed mice infected with IAV. The discrepancies observed in previous studies include CS exposure protocols, dose and duration of CS exposure (acute vs. chronic), viral dose (low and high) used, time of virus infection (ZT0-24) and duration post-infection of analyses. Regardless of these discrepancies, these studies support the fact that CS-induced inflammation plays a defining role in the initial inflammatory responses to IAV infection in the lungs. It is possible that IAV-mediated resetting of clock function in the lungs may influence the survival of mice previously exposed to chronic CS. The re-alignment of many of the clock genes following IAV infection may represent transient ‘sensitization’ of the timing system to an acute inflammatory mediator^12^,^72^. It is worth noting that, outside of rorα, only those clock genes associated with the repressive function of the clock (per1,2 and cry1,2) were in effect ‘phase-reset’ by IAV. This suggests a disparate influence of IAV infection on the oscillator, a phenomenon reported previously in response to dual entrainment by multiple cues in the liver^73^.

In conclusion, we show for the first time that IAV infection can cause temporally gated circadian disruption associated with exaggerated lung inflammation and injurious response in the lungs, culminating in exacerbations of COPD/emphysema. Our *in vivo* model of COPD exacerbation clearly demonstrates how changes in molecular clock function and immune responses can affect morbidity and mortality, rhythms of locomotor activity, lung inflammation and small airway remodeling. The role of the clock gene BMAL1 in COPD exacerbation was tested using the IAV infection model. Overall, our findings clearly show that the circadian clock plays a crucial role in modulating immune-inflammatory response during viral respiratory infection in mice. The COPD exacerbation mouse model develops augmented inflammatory responses and lung damage due to involvement of clock-dependent mechanisms that in turn affect immune response and rhythms of lung function. Understanding molecular clock function and its physiological significance in different animal models of chronic lung disease, including our COPD exacerbation model, could hasten the development of novel chronotherapeutic approaches for the treatment and management of COPD and associated exacerbations.

## Methods

All the methods were carried out in accordance with the NIH guidelines for the care and use of laboratory animals. All of the experimental protocols were approved by the University of Rochester Committee on Animal Resources (UCAR).

### Animals

Male C57BL/6J (C57) and BMAL1 knockout (KO: B6.129-Arntl^tm1Bra^/J) mice were purchased from the Jackson Laboratory (Bar Harbor, ME). C57BL/6J and BMAL1 KO mice were housed under a 12:12 light-dark (LD) cycle with lights on at 6 a.m. and fed with a regular diet and water *ad libitum* unless otherwise indicated. For chronic (6 months) CS exposure, mice were kept in a standard 12:12 L:D cycle with lights on from 6 am-6 pm throughout the experiment. Data from animals exposed to air/CS for 6 months from a previous study were included here for comparison^30^.

### Tobacco/cigarette smoke exposure and influenza A virus infection

Eight week-old mice were used for tobacco/CS exposure as previously described^30^,^74^,^75^. We used chronic (6 mo. exposure which causes pulmonary emphysema) CS exposure mouse models to determine the effect and mechanism of chronic CS exposure followed by influenza virus infection on circadian clock function and lung inflammation. Mice were exposed to CS using an environmental side-stream delivering Teague TE-10 smoking machine (Teague Enterprises, Davis, CA) for 6 months CS exposure in the Inhalation Facility at the University of Rochester Medical Center. The smoke was generated from 3R4F research cigarettes containing 11.0 mg of total particulate matter (TPM), 9.4 mg of tar and 0.73 mg of nicotine per cigarette (University of Kentucky, Lexington, KY). The total particulate matter (TPM) in per cubic meter of air in exposure chamber was monitored in real-time with a MicroDust Pro-aerosol monitor (Casella CEL, Bedford, UK), and verified daily by gravimetric sampling^30^,^74^,^75^. Control mice were exposed to filtered air in an identical chamber according to the same protocol described for CS exposure. For chronic 6 months CS exposures, 3R4F cigarettes were used to generate a mixture of sidestream smoke (89%) and mainstream smoke (11%) at a concentration of ~100 mg/m^3^ TPM, so as to avoid the possible toxicity to mice at a high concentration of long-term CS exposure^30^,^74^ according to the Federal Trade Commission protocol (1 puff/min of 2 second duration and 35 ml volume). Each smoldering cigarette was puffed for 2 seconds, once every minute for a total of 5 puffs, at a flow rate of 1.05 L/min, to provide a standard puff of 35 cm^3^. Mice received 5-hour exposures per day, 5 days/week for the duration of exposure and were sacrificed at 6-hour intervals 24h after the last CS exposure.

After 6 months chronic Air/CS exposure, mice were intranasally infected under anesthesia (Avertin; 2,2,2-tribromoethanol; Sigma-Aldrich) with 120 hemagglutination units (HAU) of influenza A virus (IAV), strain HKx31 (x31; H3N2) in 25 µl sterile PBS as previously described^32^,^76^. Mock-infected control group mice received 25 µl of sterile PBS alone. After infection, survival and body weight of all the experimental groups were monitored and recorded daily until post-infection day 9. Mice were euthanized post-infection day 9 at 6 hour intervals for 24 hours (5 time points: ZT0, ZT6, ZT12, ZT18 and ZT24). The 6-hr sampling interval was based on prior studies on circadian gene expression in mice^30^,^37^. Schematic for chronic CS exposure combined with IAV infection and IAV infection in BMAL1 KO and wild-type littermates including parameters measured from these experiments are included in the Supporting information (see Supplementary Fig. 1a-b).

### Locomotor activity recording

Mice were individually housed and allowed free access to food and water. Locomotor activity was measured using the Photobeam Activity System (San Diego Instruments, San Diego, CA), a computerized system that measures the frequency of photobeam breaks along the side of the cage. Total cage activity (photobeam break) activity was recorded in 1-min intervals and analyzed using ClockLab software (Actimetrics, Evanston IL) as previously described^30^. Circadian periodicity of locomotor activity in L:D was calculated during days 1-8 post IAV infection with a χ^2^ periodogram analysis (tau range 20–28 h).

### Real-Time Luminescence recording

Adult male Period2::luciferase knock-in (PER2::LUC) mice^77^ were euthanized three hours before lights-off (Zeitgeber Time 9–12, lights off = ZT12) by excess CO_2_ exposure. Portions of the lung were removed and collected in cold sterile Hanks balanced salt solution (HBSS). Small 5 mm^3^ fragments of lung tissue were isolated. Lung tissues were placed in 35mm culture dishes with 1.2 ml of culture medium [DMEM supplemented with B27 (Gibco), 10 mM HEPES, 352.5 μg/ml NaHCO3, 3.5 mg/ml D- glucose, 25 U/ml penicillin, 25 μg/ml streptomycin and 0.1 mM luciferin (Promega)]. Cultures were prepared with clean media as described above (control) or the same media containing cigarette smoke extract (CSE 0.1%), media containing 300 HAU influenza A virus ( and sealed with sterile vacuum grease and a glass coverslip. Sealed cultures were maintained at 35 °C in a light-tight incubator and luminescence was continuously recorded (counts/sec) with an automated luminometer (LumiCycle, Actimetrics). Raw luminescence data were detrended (24 h moving average) and smoothed (2 h moving average; Origin Pro 8.5, OriginLabs, Northampton, MA) as previously described^30^.

### Bronchoalveolar lavage (BAL)

Mice were anesthetized at 24 h after the last exposure or on day 9 post-infection by an intraperitoneal injection with 100 mg/kg (BW) of pentobarbital sodium (Abbott Laboratories, Abbott Park, IL) and sacrificed by exsanguination. The heart and lungs were removed *en bloc*, and the lungs were lavaged three times with 0.6 ml of saline (0.9% sodium chloride) via a cannula inserted into the trachea as described previously^75^,^78^. The lavaged fluid was centrifuged, and the cell-free supernatants were frozen at −80°C for later analysis. The BAL inflammatory cell pellet was resuspended in 1 ml saline and the total cell number was counted using a hemocytometer. Cytospin slides (Thermo Shandon, Pittsburgh, PA) were prepared using 50,000 cells per slide, and differential cell counts (~500 cells/slide) were performed on cytospin-prepared slides stained with Diff-Quik (Dade Behring, Newark, DE).

### Proinflammatory mediators analysis

The levels of proinflammatory mediators, such as CCL2/monocyte chemotatic protein 1 (MCP-1), CXCL2/macrophage inflammatory protein 2 (MIP-2), interleukin 6 (IL-6) and TGF-β1 in bronchioalveolar lavage fluid were measured by enzyme-linked immunosorbent assay (ELISA) using respective duo-antibody kits (R&D Systems, Minneapolis, MN) according to the manufacturer’s instructions. The results were expressed in the samples as pg/ml.

### Histological analysis, Periodic Acid-Schiff (PAS) staining, immunohistochemistry and trichrome staining

Fixed tissues were H&E stained for inflammation scoring and mean linear intercept analysis. Histological analysis of H&E stained slides were used to determine bronchial inflammation using a semi-quantitative method. Briefly, the intensity of bronchial inflammation was scored on a scale of 1 to 9. 0, for no inflammation; 1–3 for scant cells but not forming a defined layer; 4–6, for one to three layers of cells surrounding the vessels; 7–9, for four or greater layers of cells surrounding the vessel or bronchial area. For each treatment multiple lung lobes from n = 4-5 slide/group were scored and average values were presented as bronchial inflammation scores.

Similarly airway mucus was identified by the Periodic Acid-Schiff (PAS) staining (Sigma-Aldrich, St. Louis, MO) and PAS positive cells were quantified by a semi-quantitative method with slight modification as previously described^61^. In brief, airways were examined under light microscopy and assigned a score between 0 and 3 based on the following criteria: 0, for no staining; 1, for PAS staining <25% of airway perimeter; 2, for PAS staining 25 to <50% of airway perimeter; and 3, for PAS staining >50% of airway perimeter. Mucus scores were obtained by scoring 3–4 different areas per slide from n = 4-5 slide/group. The average from all the areas scored per each treatment group was used to calculate the percentage of PAS positive cells.

Immunostaining was performed on formalin-fixed, paraffin-embedded lung tissue. Paraffin sections (4 µm thick) were deparaffinized and then rehydrated through series of xylene and graded ethanol. Antigen retrieval was performed by heating in citrate buffer (10 mM Citric acid, 0.05% Tween 20, pH 6.0). Primary antibody was incubated overnight at 4°C with rabbit anti-CCSP polyclonal antibody (Seven Hills Bioreagents, Cincinnati, OH) and mouse anti α-smooth muscle actin (Abcam, Cambridge, MA) antibodies^32^,^74^,^76^,^79^. Appropriate fluorescently labeled secondary antibodies (FITC- or Texas red-conjugated 2° antibodies) were used to detect the immune complexes before tissues sections were counterstained with 4’,d-diamidino-2-phenylindole (dapi).

Gomori’s trichrome staining was performed according to the manufacturer’s instructions (Richard-Allan Scientific, Kalamazoo, MI). The nuclei stains black, cytoplasm and muscle fibers in red and the collagen deposition stains blue. Both double immunostaining and trichrome stained tissue sections were visualized with Nikon Eclipse Ni-U fluorescence microscope (Nikon, Melville, NY) and images were captured with a SPOT-RT3 digital camera (Diagnostic Instruments, Sterling Heights, MI). Quantification of fibrosis was done using the Ashcroft scoring system^80^.

### Lung morphometry

Mouse lungs (which had not been lavaged) were inflated by 1% low-melting point agarose at a pressure of 25 cm H_2_O, and then fixed with 4% neutral buffered formalin^74^,^75^. Fixed lung tissues were dehydrated, embedded in paraffin and sectioned (4 μm) using a rotary microtome (MICROM International GmbH). Lung sections were deparaffinized and rehydrated by passing through a series of xylene and graded ethanol, then stained with hematoxylin and eosin (H&E). Alveolar size was estimated from the mean linear intercept (Lm) of the airspace, which is a measure of airspace enlargement/emphysema using the MetaMorph software (Molecular Devices) as previously described^30^,^74^,^75^. Lm was calculated for each sample based on 10 random fields per slide observed at a magnification of ×200. The airway and vascular structures were eliminated from the analysis.

### Measurements of lung mechanical properties

Lung mechanical properties in mouse lung were determined using Scireq Flexivent apparatus (Montreal, Canada) as described previously^30^,^74^,^75^. Briefly, lung compliance (C), lung resistance (R), and tissue elastance (E) were measured in mice, anesthetized by sodium pentobarbital (50 mg/kg BW, intraperitoneally). A tracheotomy was performed, and an 18-gauge cannula was inserted 3 mm into an anterior nick in the exposed trachea and connected to a computer controlled rodent ventilator (FlexiVent; SCIREQ). Initially, the mice were ventilated with room air (150 breaths/min) at a volume of 10 ml/kg body mass. After 3 min of ventilation, measurement of lung mechanical properties was initiated by a computer-generated program to measure quasi-static compliance, lung resistance, and tissue elastance at 3 cm H_2_O positive end expiratory pressure obtained by fitting a model to each impedance spectrum. The calibration procedure removed the impedance of the equipment and tracheal tube within this system^81^. These measurements were repeated three times for each animal using Scireq FlexiVent apparatus (Montreal, Canada) as described previously^30^,^74^,^75^,^79^.

### RNA isolation and quantitative PCR

Total RNA was isolated from non-lavaged lung tissue specimens (stored in RNAlater, Ambion, Austin, TX) using RNeasy kit (Qiagen, Valencia, CA). RNA yields were determined by UV absorbance using a Nanodrop instrument (ND-1000 Spectrophotometer, NanoDrop Technologies). cDNA was synthesized from 0.5 μg of total RNA using the RT^2^ First Strand Kit (SABioscience, Frederick, MD). To validate the expression of diverse genes in lung tissue by quantitative real-time PCR (qPCR) (Bio-Rad CXF-96 real-time system) using the SYBR Green qPCR Master mix from SABioscience. In chronic Air/CS exposed mice infected with or without influenza A virus, this includes qPCR data from circadian genes at ZT0-ZT24 time point (n = 3-4 mice/group) in all datasets. All the specific primers were purchased from SABioscience. Expression of genes was normalized to RPL13 (60S ribosomal protein L13 gene) levels. The samples from chronic air- and CS-exposed mice represented in this study were obtained from our previous study that was conducted in parallel with the chronic air and CS combined with influenza A virus infection^30^. In chronic air exposure group, qPCR data gathered from circadian gene expression at the ZT24 time point (n = 2/air group) in all qPCR datasets. Relative RNA abundance was quantified by the comparative 2^-ΔΔCt^ methods. Significant rhythms of gene expression were verified using CircWave software (Version 1.4). In addition, the center of gravity (COG) or peak phase was determined for each rhythm using CircWave as previously described^30^,^82^.

### Statistical analysis

The period of PER2::LUC expression in each tissue explant was determined using a chi-squared periodogram analysis (LumiCycle Analysis Software, Actimetrics). A minimum of 5 days of data were used to calculate the period of PER2::LUC expression in each explant of lung tissue. Period data from lung tissue were analyzed with two-factor ANOVA. Data are representative of mean ± SEM. For statistical analysis of qPCR data, CircWave software (Version 1.4) was employed. In addition to multiple non-linear regression analyses to determine if the data conform to a significant rhythm, CircWave also calculates the peak of gene expression or Center of Gravity (COG). The software determines a peak of gene expression even if the qPCR data do not conform to a significant circadian rhythm (*P* < 0.05). In addition to CircWave analysis, statistical significance between air-exposed, Air+Virus and CS+Virus groups was calculated by Fisher’s multiple comparisons using two-way ANOVA. To emphasize the waveform of the data, percentage of daily activity (light and dark phase) was subjected to nonlinear regression analysis with a 6^th^ order polynomial using GraphPad (Prism 6). Statistical analysis of significance was calculated using one-way Analysis of Variance (ANOVA) followed by Tukey’s *post-hoc* test for multi-group comparisons using StatView software. Mortality was evaluated by comparison of survival curves and analyzed for significance by Log-rank (Mantel-Cox) test using GraphPad (Prism 6). *P* < 0.05 was considered as significant.

## Author Contributions

I.K.S., I.R., M.T.S., and B.P.L. conceived and designed the experiments; I.K.S., T.A., H.Y., J.H., J.G., and M.T.S. performed the experiments; I.K.S., J.H., M.T.S. data analysis; I.K.S., T.A., H.Y., J.H., M.T.S., B.P.L., and I.R. interpretation of results, editing the manuscript; I.K.S. M.T.S., and I.R. PER2::luciferase expression assays; I.K.S., M.T.S., and I.R. wrote the paper.

## Supplementary Material

Supplementary Information Supplementary Information - Suppl. Figs 1-10 and Suppl. Tables 1-3

## Figures and Tables

**Table 1:** Center of gravity (COG) values and significance for each clock gene and other gene expression rhythm as determined by CircWave analysis in chronic air- and CS-exposed mice infected with influenza A virus

Clock gene	COG of Air+Virus ± SD	*P-* value for Air+Virus	COG of CS+Virus ± SD	*P-* value for CS+Virus
Bmal1	6.809 ± 2.219	0.0002***	6.803 ± 1.763	0.013**
Clock	0.807 ± 2.617	NS	5.569 ± 2.829	NS
Per1	2.582 ± 2.368	0.024*	13.792 ± 3.503	NS
Per2	3.577 ± 1.967	0.034*	20.847 ±2.698	NS
Cry1	3.841 ± 2.201	0.038*	21.688 ± 2.682	NS
Cry2	1.057 ± 2.500	NS	10.463 ± 3.315	NS
Rev-erbα	21.883 ± 2.4961	NS	16.362 ± 2.973	NS
Rev-erbβ	4.507 ± 1.907	0.037*	2.296 ± 2.686	NS
Rorα	2.130 ± 2.666	NS	21.104 ± 3.095	NS
Other genes				
Sirt1	3.599 ± 2.383	0.041*	2.054 ± 3.118	NS
Ahr	0.919 ± 2.014	NS	0.908 ± 3.115	NS
Muc5 ac	13.563 ± 3.089	NS	13.190 ± 3.671	NS

Data are shown as mean ± SEM (n = 3-4 per group). *P < 0.05, **P < 0.01, ***P < 0.001, significance of rhythmicity as determined by CircWave analysis in Air+Virus or CS+Virus exposed mice. NS, Not significant

**Table 2:** Center of gravity (COG) values and significance for rhythm of lung mechanical properties as determined by CircWave analysis in chronic air alone, air- and CS-exposed mice infected with influenza A virus

Mechanical Properties	Air‡		Air+Virus		CS+Virus	
	COG ± SD	*P-* value	COG ± SD	*P- value*	COG± SD	*P-* value</emph>
Compliance	0.64 ± 3.01	0.001***	15.08 ± 3.61	0.005**	3.00 ± 3.11	0.014**
Resistance	23.35 ± 3.04	NS	1.76 ± 3.59	0.0001***	15.45 ± 3.70	0.005**
Elastance	23.35 ± 3.08	0.001***	3.84 ± 3.59	0.004**	23.51 ± 3.39	0.023*

Data are shown as mean ± SEM (n = 3-4 per group). *P < 0.05, **P < 0.01, ***P < 0.001, significance of rhythmicity as determined by CircWave analysis in air or Air+Virus or CS+Virus exposed mice. NS, Not significant

‡Data from animals exposed to air for 6 months from a previous study were included here for comparison^30^.

**Table 3:** Lung mechanical properties measured during different ZTs in WT mice exposed to chronic air and CS infected with influenza A virus

Sl. No.	ZT time (Hrs)	Lung Compliance (mL/cmH_2_O)		Lung Resistance (cmH_2_O.s/mL)		Tissue Elastance (cmH_2_O/mL)	
		Air+Virus	CS+Virus	Air+Virus	CS+Virus	Air+Virus	CS+Virus
1.	ZT6	0.038 ± 0.003	0.052 ± 0.003 $	0.849 ± 0.018	0.683 ± 0.042	26.943 ± 1.775	19.587 ± 1.390
2.	ZT12	0.054 ± 0.002	0.041 ± 0.007 **, †	0.673 ± 0.032	0.972 ± 0.181*, †	18.704 ± 0.643	27.801 ± 6.493*
3.	ZT18	0.047 ± 0.003	0.036 ± 0.012**, ††	0.748 ± 0.016	1.171 ± 0.365*, §, ††	21.464 ± 1.259	32.189 ± 11.074 §, †
4.	ZT24	0.048 ± 0.001	0.035 ± 0.004 **, ††	0.809 ± 0.016	0.783 ± 0.078 #	21.022 ± 0.337	28.697 ± 3.123

Data are shown as mean ± SEM (n = 2–4 per group). **P < 0.01, significant compared to Air+Virus (ZT12); ^$^ *P* < 0.05, significant compared to Air+Virus (ZT6); *P < 0.05, significant compared to Air+Virus (ZT12, and ZT18); ^§^ *P* < 0.05, significant compared to Air+Virus (ZT12 and ZT24); ^†^ *P* < 0.05, and ^†† ^*P* < 0.01, significant compared to CS+Virus (ZT6);^ # ^*P* < 0.05, significant compared to CS+Virus (ZT18).
