# Supplementary material for: Influenza A virus-dependent remodeling of pulmonary clock function in a mouse model of COPD
Source: Sci Rep. 2015 Apr 29;4:9927. doi: 10.1038/srep09927 (PMC4413879; doi:10.1038/srep09927)
Supplement: Supplementary Information — Suppl. Figs 1-10 and Suppl. Tables 1-3 [file srep09927-s1.pdf]

Supplementary Information

Influenza A virus-dependent remodeling of pulmonary clock function in a mouse model of COPD

Isaac K. Sundar, Tanveer Ahmad, Hongwei Yao, Jae-woong Hwang, Janice Gerloff,

B. Paige Lawrence, Michael T. Sellix, and Irfan Rahman

Supplementary Figures

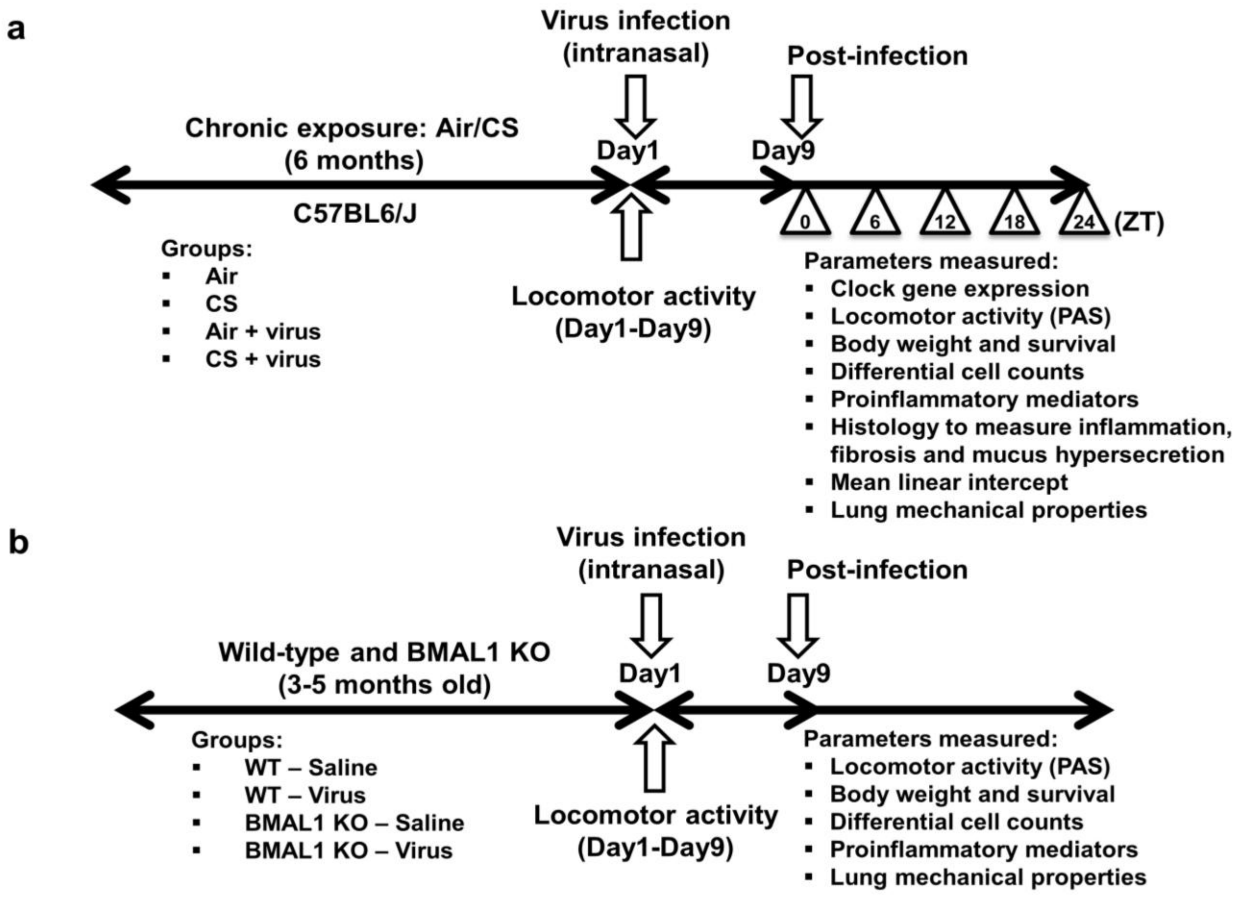

Supplementary Fig. 1. Experimental design for chronic air or CS exposure combined with influenza A virus (IAV) infection (COPD-exacerbation model) or IAV infection alone. (a) Mouse model of COPD exacerbation by chronic CS followed by IAV infection. C57BL/6J mice were exposed to chronic air (control) or CS was infected with a single dose of influenza A virus. **(b)** Mouse model of influenza A virus infection in wild-type (WT) and BMAL1 knockout (KO) mice. WT and BMAL1 KO mice (about 3-5 months old) were infected with single dose of influenza A virus. For both experiments locomotor activity, body weight and survival were recorded prior to and during IAV infection (Post-infection day 1-9). Locomotor activity was also recorded for several days prior to infection in WT and BMAL1 KO mice. Rhythms of lung function were measured on day 9 post-infection. Collection of lung tissue for clock gene expression and histological analyses and BAL fluid collection was also conducted on day 9 post-infection.

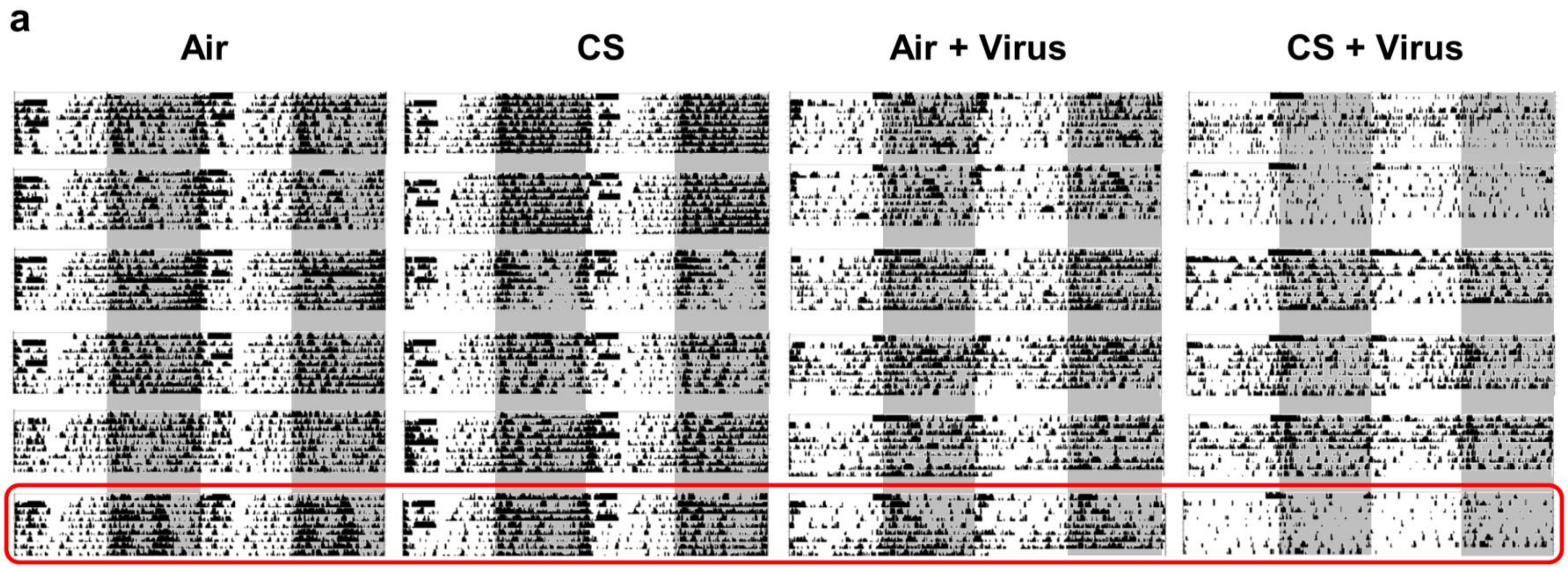
a
Air
CS
Air + Virus
CS + Virus

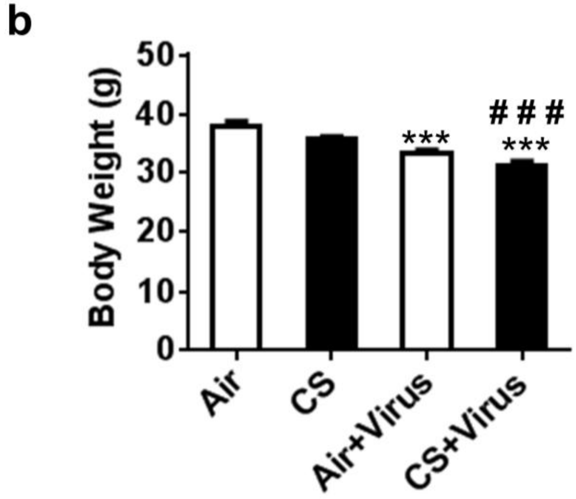
b
Body Weight (g)
50
40
30
20
10
0
***
###
***
Air
CS
Air+Virus
CS+Virus

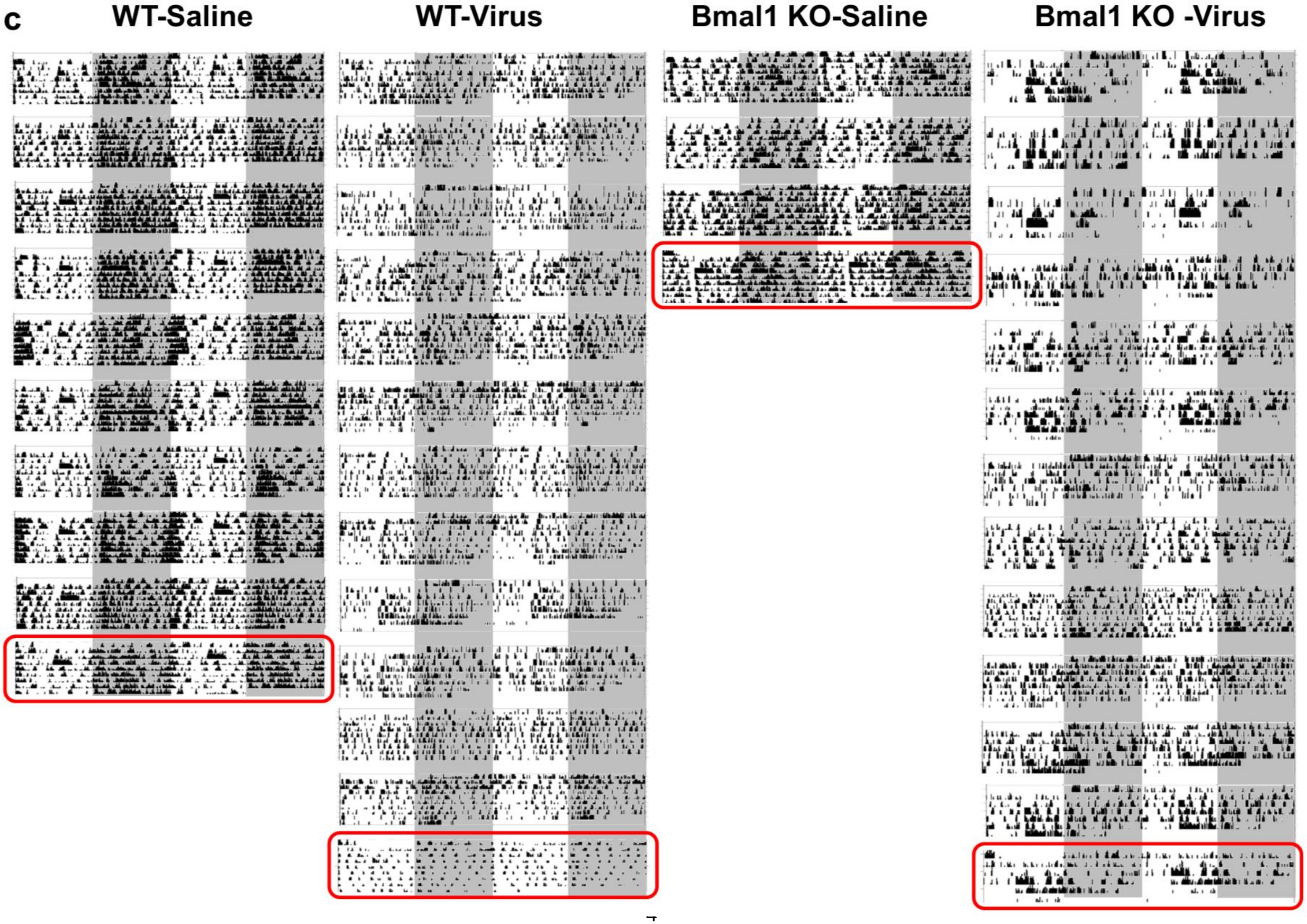
c
WT-Saline
WT-Virus
Bmal1 KO-Saline
Bmal1 KO -Virus

Supplementary Fig. 2. Locomotor activity during Influenza A virus infection in mice. Data from chronic (6 months) air- or CS-exposed mice given intranasal inoculation of either saline (control group) or influenza A virus (IAV; treatment group) at ZT4-6 are shown. **(a)** Double plotted actograms of total cage activity over 9 days from chronic air- and CS-exposed mice, and IAV infected air- and CS-exposed mice from Day 0 to day 9 post-infection. **(b)** Body weight of chronic air, CS, Air+Virus and CS+Virus groups were measured at the end of exposure or day 9 post IAV infection in all the treatment groups. Data are mean ± SEM. *** $P < 0.001$ significant compared to air- or CS-exposed mice; $^{\#\#\#}$ $P < 0.001$; significant compared to air-exposed mice. **(c)** Double plotted actograms of total cage activity from WT and BMAL1 KO mice given intranasal inoculation of saline (control group) or influenza A virus (IAV; treatment group) from Day 0 to day 9 post-infection. In panels **a** and **c**, gray shading indicates the dark phase (ZT12-24). The actograms marked by red squares are those presented in the main figures of the manuscript.

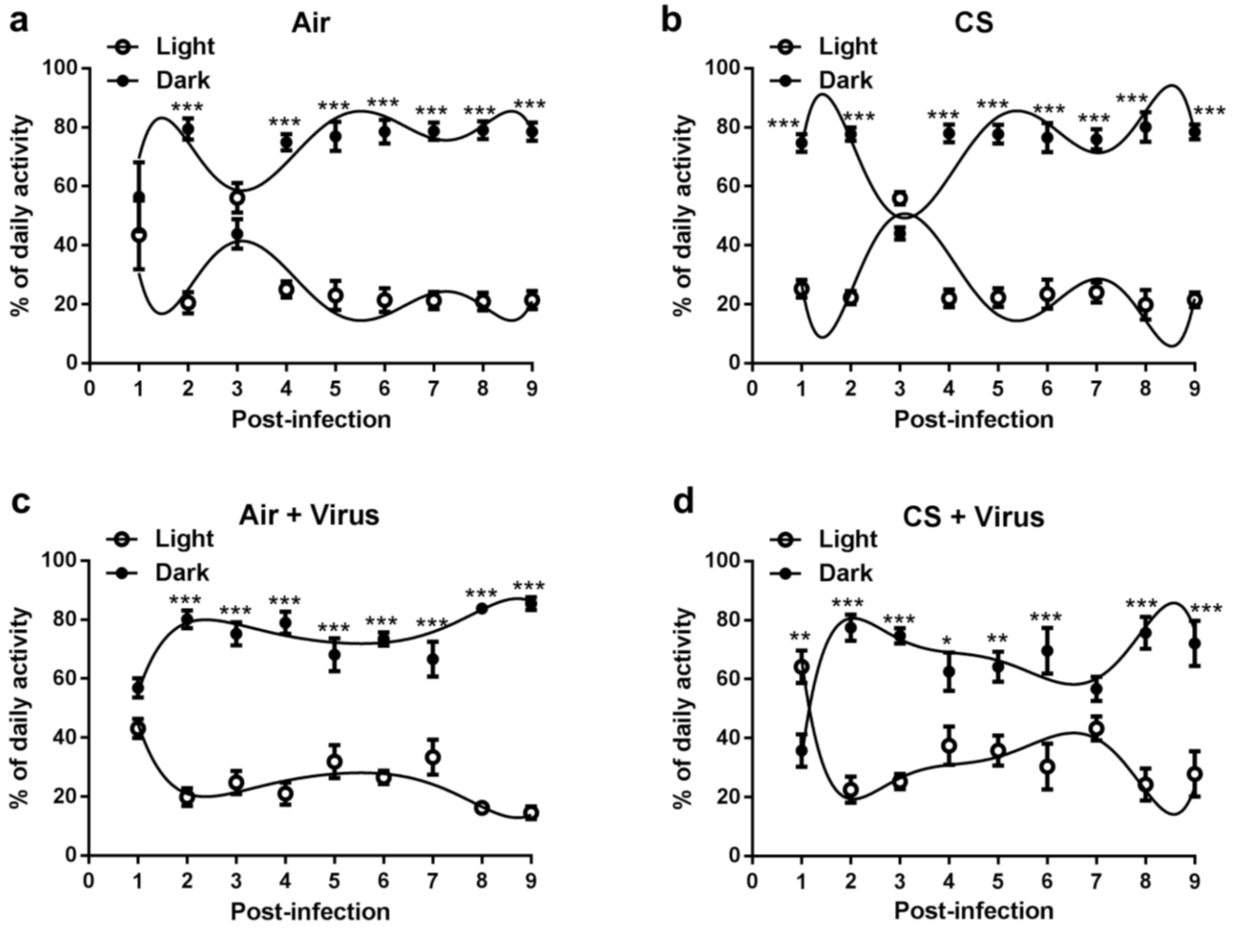

Supplementary Fig. 3. Distribution of daily activity in chronic Air, CS, Air+Virus and CS+Virus exposed mice. Data from chronic (6 months) air- or CS-exposed mice given intranasal inoculation of either saline (control group) or influenza A virus (IAV; treatment group) at ZT4-6 are shown. Locomotor activity data were recorded from day 1-9 post-infection. Data are plotted as percentage of daily activity as a function of photoperiod (12h light phase; open circles, 12h dark phase; closed circles). **(a)** Chronic air-exposed mice were primarily active (70-80% activity) during the dark phase and less active (20-30%) during the light phase from day 2-9 post-saline infusion. **(b)** Chronic CS-exposed mice showed overall greater activity (70-80%) during the dark phase, with the exception being a transient inversion of this relationship on day 3 of recording similar to air-exposed mice. **(c)** Chronic air-exposed mice infected with IAV were primarily active (70-80% activity) during the dark phase and less active (20-30%) during the light phase from day 2-9 post-infection similar to air-exposed mice. **(d)** As in air-exposed mice, IAV infection of chronic CS-exposed mice transiently reduced nighttime activity and increased

daytime activity on day 1 post-infection. By day 2, this response was reversed but the level of nighttime activity gradually declined in parallel with a slight increase in daytime activity over the next 4-5 days after infection such that a near 50/50 split was reached by day 7. By day 8-9 post-infection the CS+Virus group appeared to recover their normal nighttime (70-80%) and daytime (20-30%) activity levels, most likely due to mortality among mice with a more even distribution of activity. Data are mean ± SEM (n=6 mice/group). *P < 0.05; **P < 0.01; ***P < 0.001 significant compared to respective light phase in each treatment groups.

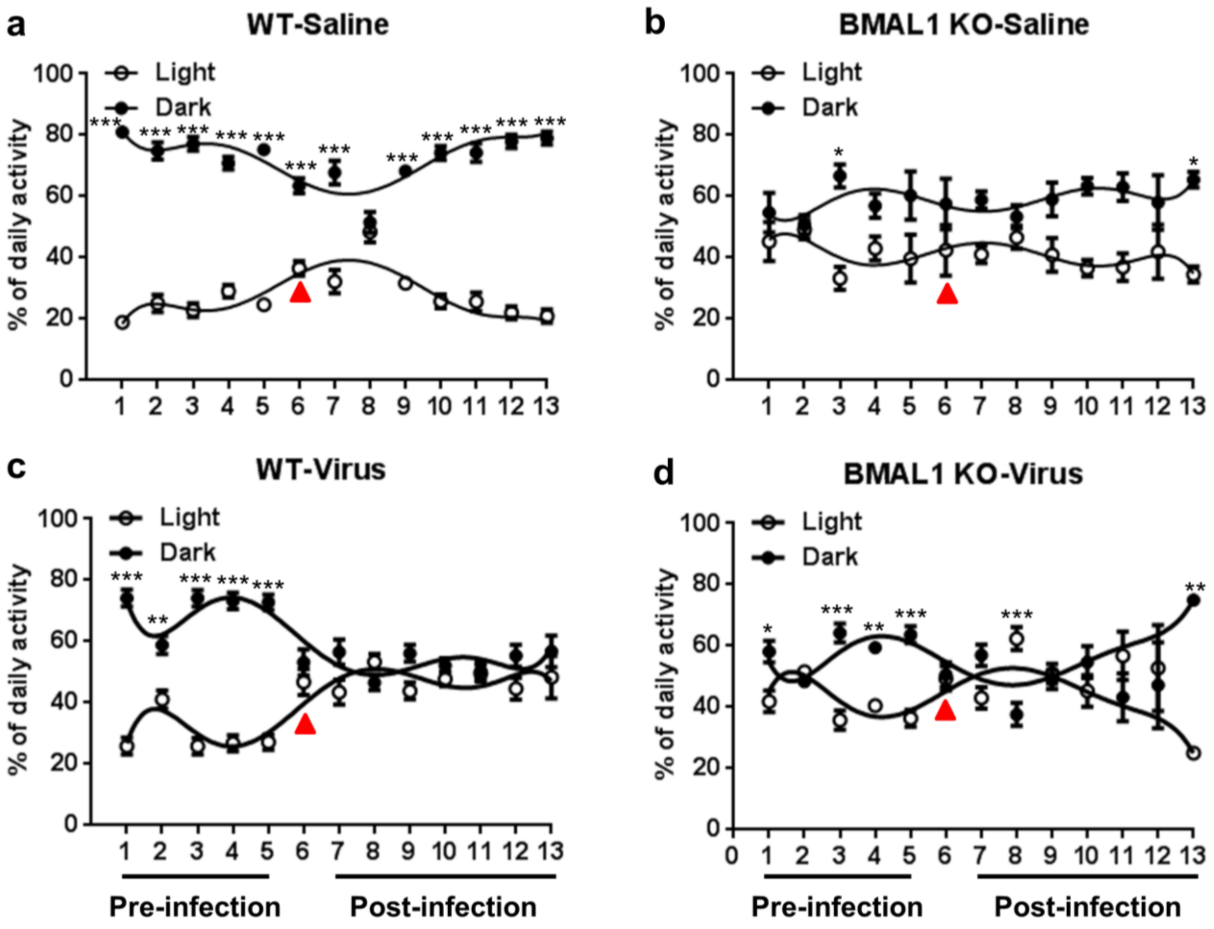

Supplementary Fig. 4. Distribution of daily activity in WT and BMAL1 KO mice before and after IAV infection. Data from WT and BMAL1 KO mice given intranasal inoculation of either saline (control group) or influenza A virus (IAV; treatment group) at ZT4-6 are shown. Locomotor activity was recorded from individual mice 5 days prior to IAV infection and for 7 days post-infection. Data are plotted as percentage of daily activity as a function of photoperiod (12h light phase; open circles, 12h dark phase; closed circles). **(a)** Saline-treated WT mice were primarily (70-80% activity) active during the dark phase both prior to and after saline infusion. **(b)** In contrast with WT mice, uninfected BMAL1 KO mice displayed a near even distribution of activity across the light-dark cycle that was not affected by saline infusion. This distribution is due to behavioral arrhythmia in these mice **(c)** IAV infection significantly reduced the level of nighttime activity and increased daytime activity in WT mice, resulting in a near even distribution of activity across the 24h day. **(d)** IAV infection further enhanced the amount of daytime activity and reduced the level of nighttime activity in BMAL1 KO mice, an effect also

associated with near complete arrhythmicity. Data are representative of mean ± SEM (n=10 WT-Saline; n=4 BMAL1 KO-Saline; n=13 WT-Virus; n= 13 BMAL1 KO-Virus). In both A and B the day of IAV or saline infusion is indicated by a red arrowhead. *$P < 0.05$; **$P < 0.01$; ***$P < 0.001$ significant compared to corresponding light phase in each treatment group.

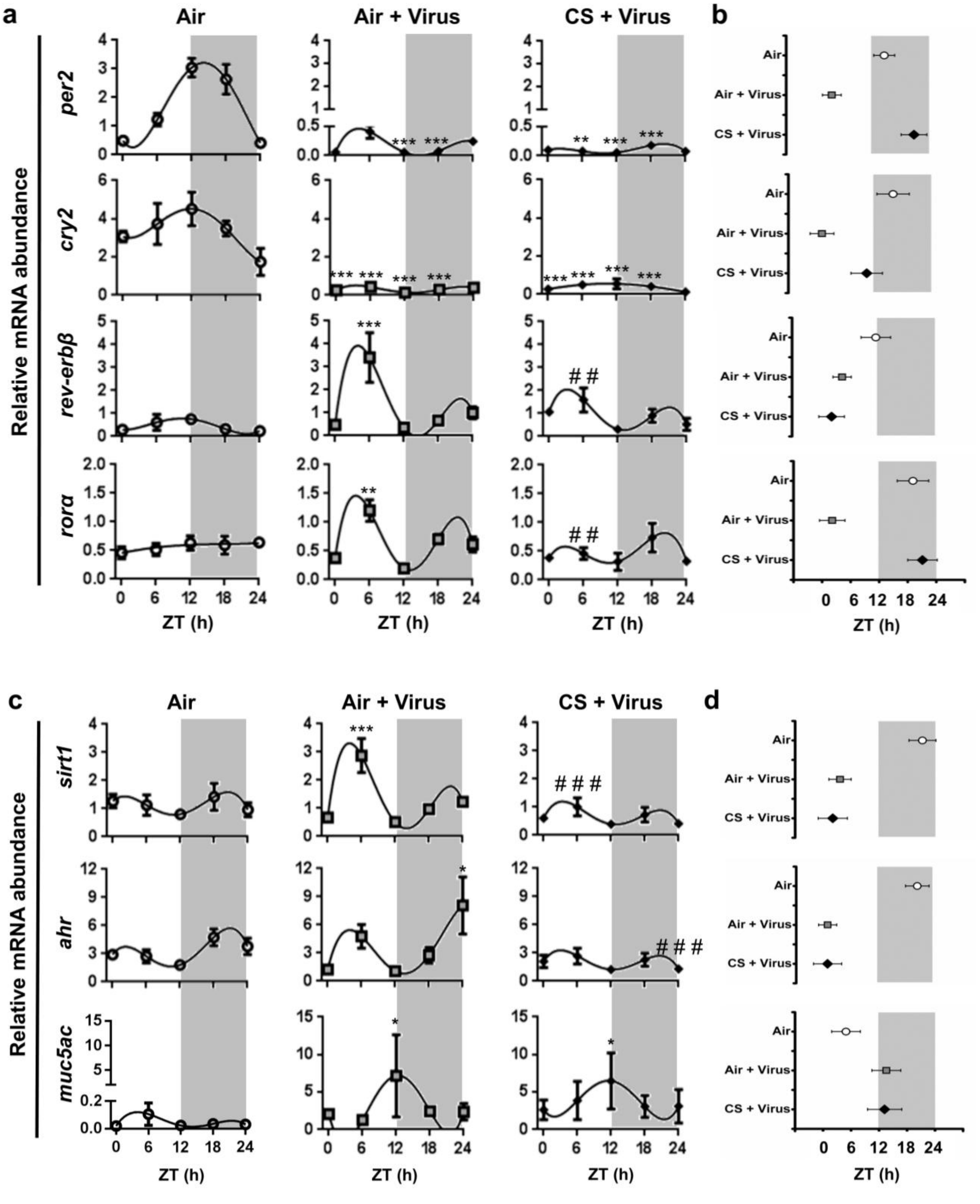

Supplementary Fig. 5. Diurnal rhythms of clock gene and clock-controlled gene expression in the lungs are differentially affected by chronic CS and IAV infection. Data from chronic

(6 months) air- or CS-exposed mice given intranasal inoculation of either saline (control group) or influenza A virus (IAV; treatment group) at ZT4-6 are shown. Data from uninfected air-exposed mice from a previous experiment were included for comparison [panels a-d] [30]. **(a)** Expression of core clock genes (*per2, cry2, rev-erbβ* and *rorα*) in lung tissue was determined with qPCR. CircWave analysis confirmed statistically significant rhythms of clock gene expression in lung tissue from the Air+Virus ($P < 0.05$ for *per2* and rev-erbβ) but not for *per2, cry2, rev-erbβ* and *rorα* in CS+Virus group. **(b)** The peak phase of each clock gene was determined with CircWave and plotted on a horizontal phase map. Gray shading indicates the dark phase (ZT12-24). Data from air-exposed (open circle), Air+Virus (gray square) and CS+Virus (solid diamond) mice are shown as mean ± SEM (n=3-4 mice/group) for each time point. ** $P < 0.01$; *** $P < 0.001$ significant compared to Air group. $^{\#\#}$ $P < 0.01$; significant compared to Air+Virus group. **(c)** Chronic CS exposure combined with influenza A virus infection altered rhythms of *sirt1, ahr* and *muc5ac* gene expression in mouse lungs. CircWave analysis confirmed statistically significant rhythms of *sirt1* ($P < 0.05$) but not *ahr* or *muc5a* in the Air+Virus group and did not detect any significant rhythms (*sirt1, ahr* and *muc5ac*) in the CS+Virus group. **(d)** Peak phase of gene expression for each gene (even in the absence of statistically significant rhythmicity) was plotted on a horizontal phase map. Gray shading indicates the relative dark phase (ZT12-24). Data from air-exposed (open circle), Air+Virus (gray square) and CS+Virus (solid diamond) mice are representative of mean ± SEM (n=3-4 mice/group) for each time point. * $P < 0.05$; *** $P < 0.001$ significant compared to Air group; $^{\#\#\#}$ $P < 0.001$; significant compared to Air+Virus group.

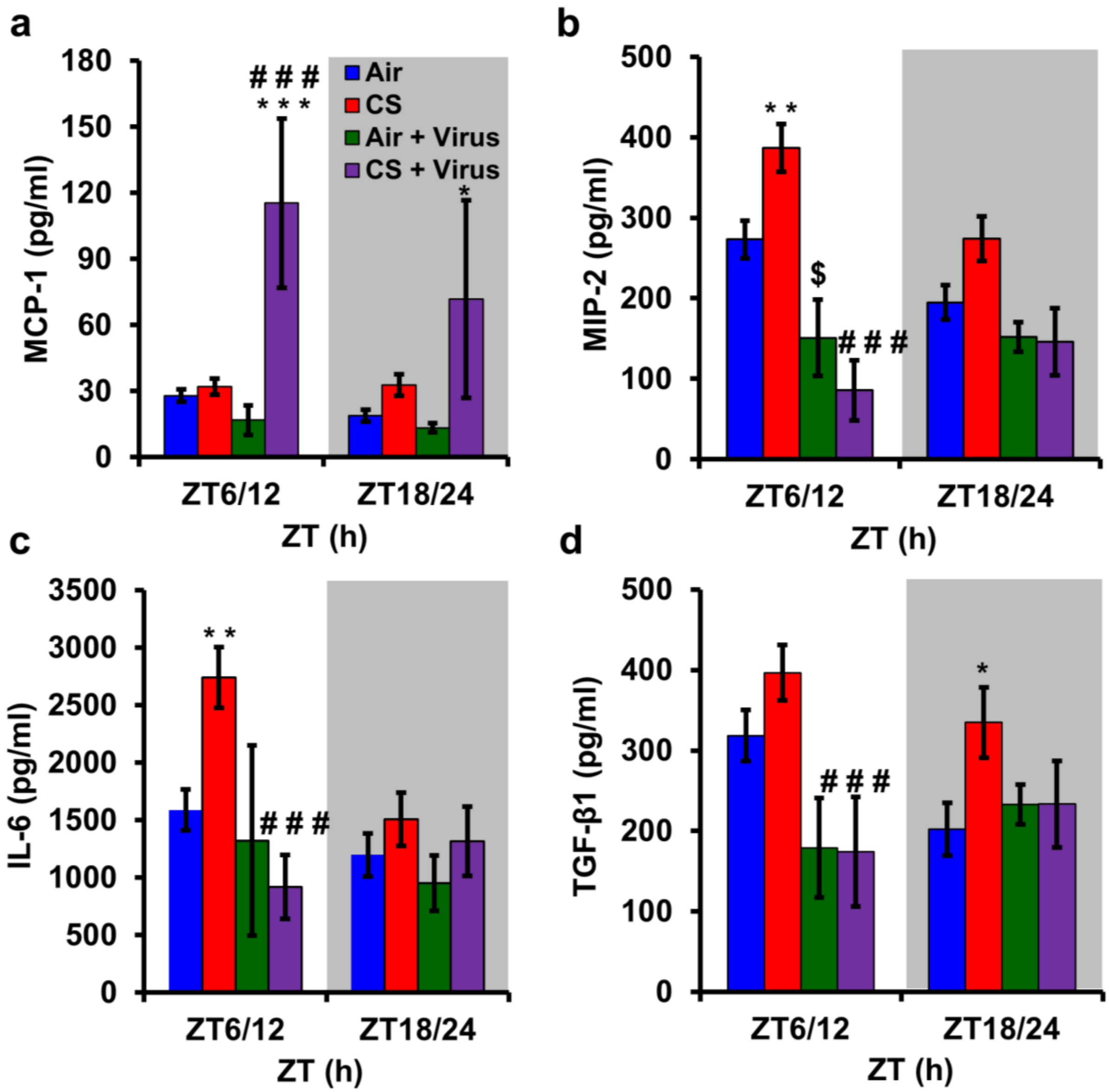

Supplementary Fig. 6. Influenza A virus infection has time-dependent effects on proinflammatory cytokine release in lungs from mice exposed to chronic CS. Data from chronic (6 months) air- or CS-exposed mice given intranasal inoculation of either saline (control group) or influenza A virus (IAV; treatment group) at ZT4-6 are shown. After day 9 post-infection, BAL fluid collected from harvested mouse lungs every 6 h for 24 h. Levels of proinflammatory mediators **(a)** MCP-1, **(b)** MIP-2, **(c)** IL-6 and **(d)** TGF-β1 were measured in BAL fluid obtained from air, CS, Air+Virus and CS+Virus exposed mice. Both the daytime

(ZT6+ZT12) and nighttime (ZT18+ZT24) measurements for proinflammatory cytokine release were combined for the analysis. Data are representative of mean ± SEM (n = 8-10 air; n = 12-14 CS; n= 4-7 Air+Virus and n=4-7 CS+Virus) for each time point (ZT6/12 and ZT18/24). * P < 0.05; ** P < 0.01; *** P < 0.001, significant compared to air or Air+Virus; $^{\$}$ P < 0.05 significant compared to air-exposed mice; $^{###}$ P < 0.001 significant compared to CS-exposed mice.

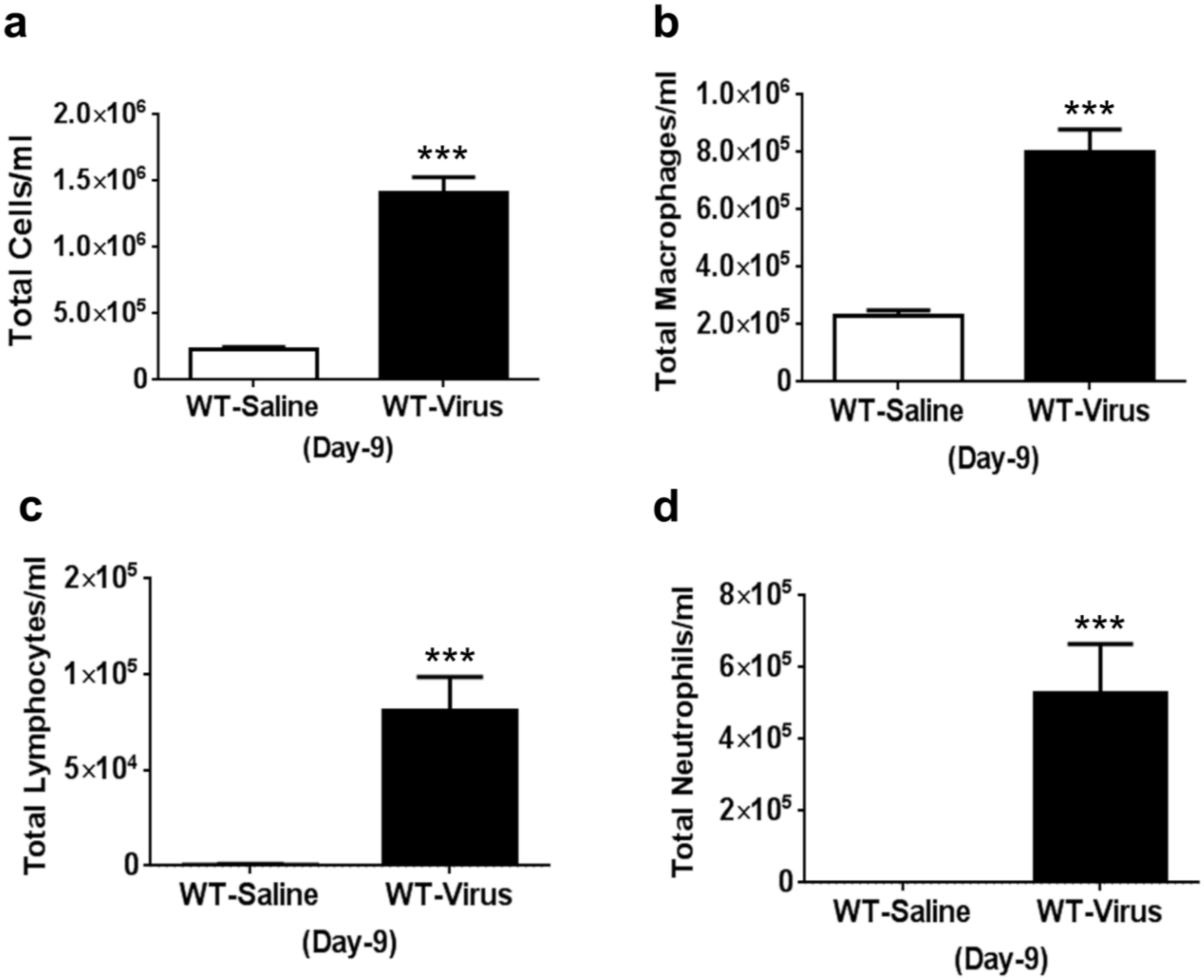

Supplementary Fig. 7. Influenza A virus infection increases inflammatory cell influx in mouse lungs. Data from WT mice given intranasal inoculation of either saline (control group) or influenza A virus (IAV; treatment group) at ZT4-6 are shown. The total number of cells in BAL fluid from WT-Saline and WT-Virus infected mice was determined day 9 post-infection. At least 500 cells in the BAL fluid were counted on cytospin slides stained with Diff-Quik to determine the **(a)** total cells, **(b)** total macrophages, **(c)** total lymphocytes and **(d)** total neutrophils. Data are representative of mean ± SEM (n=9 WT-Saline and n=7 WT-Virus group). *** $P < 0.001$ significant compared to WT-Saline.

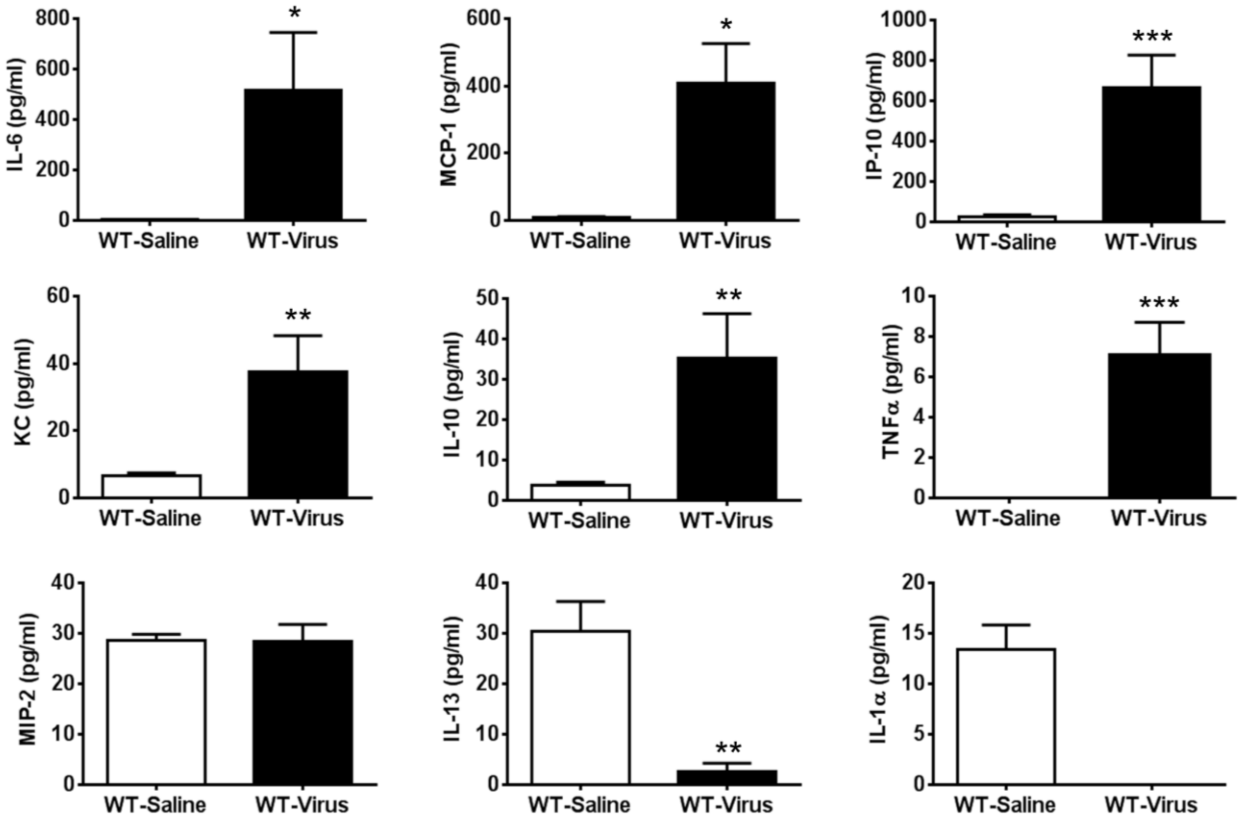

Supplementary Fig. 8. Influenza A virus infection increases proinflammatory cytokine release in WT mouse lungs. Data from WT mice given intranasal inoculation of either saline (control group) or influenza A virus (IAV; treatment group) at ZT4-6 are shown. After day 9 post-infection, BAL fluid collected from harvested mouse lungs every 6 h for 24 h. Levels of proinflammatory mediators were measured using milliplex map kit (Mouse cytokine/chemokine magnetic bead panel - 12 plex) in BAL fluid obtained from WT-Saline and WT-Virus infected mice. Data are representative of mean ± SEM (n=4-9 WT-Saline and n=7 WT-Virus). * $P < 0.05$; ** $P < 0.01$; *** $P < 0.001$ significant compared to WT-Saline.

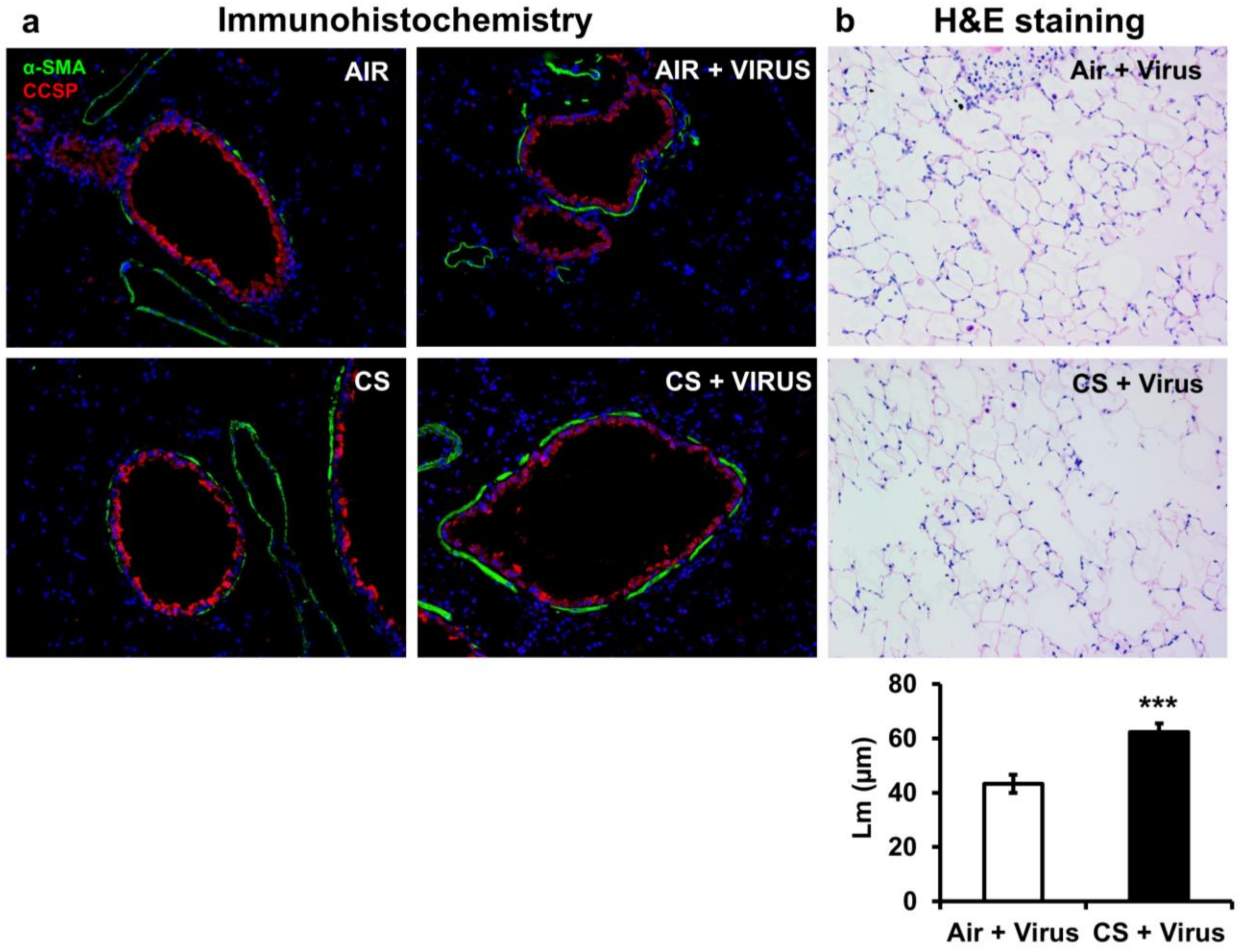

Supplementary Fig. 9. Immunohistochemisty and morphometric analysis in chronic CS-exposed mice infected with influenza A virus. Data from chronic (6 months) air- or CS-exposed mice given intranasal inoculation of either saline (control group) or influenza A virus (IAV; treatment group) at ZT4-6 are shown. Alpha-smooth muscle actin (α-SMA) and Clara cell secretory protein (CCSP) expression in activated myofibroblasts and airway epithelial cells, respectively. At day 9 post-infection the levels of α-SMA indicating increase in activated myofibroblasts and reduced CCSP expression in the airway epithelium of chronic CS-exposed mice infected with influenza A virus infection. **(a)** Representative images of α-SMA expressing fibroblasts (green color) and CCSP-expressing airway epithelial cells (red color) followed by counterstaining with 4',6-diamidino-2-phenyl-indole (DAPI, blue) in air, CS, Air+Virus and

CS+Virus infected mice. Original magnification x200 (n=2-3 mice/group). **(b)** Representative images of hematoxylin and eosin (H&E) stained lung sections from Air+Virus and CS+Virus infected mice. Original magnification: x200. Mean linear intercept (Lm) was calculated from H&E stained images using MetaMorph software (Molecular Devices). Data are representative of mean ± SEM (n=12-14 mice/group). *** $P < 0.001$; significant compared to Air+Virus.

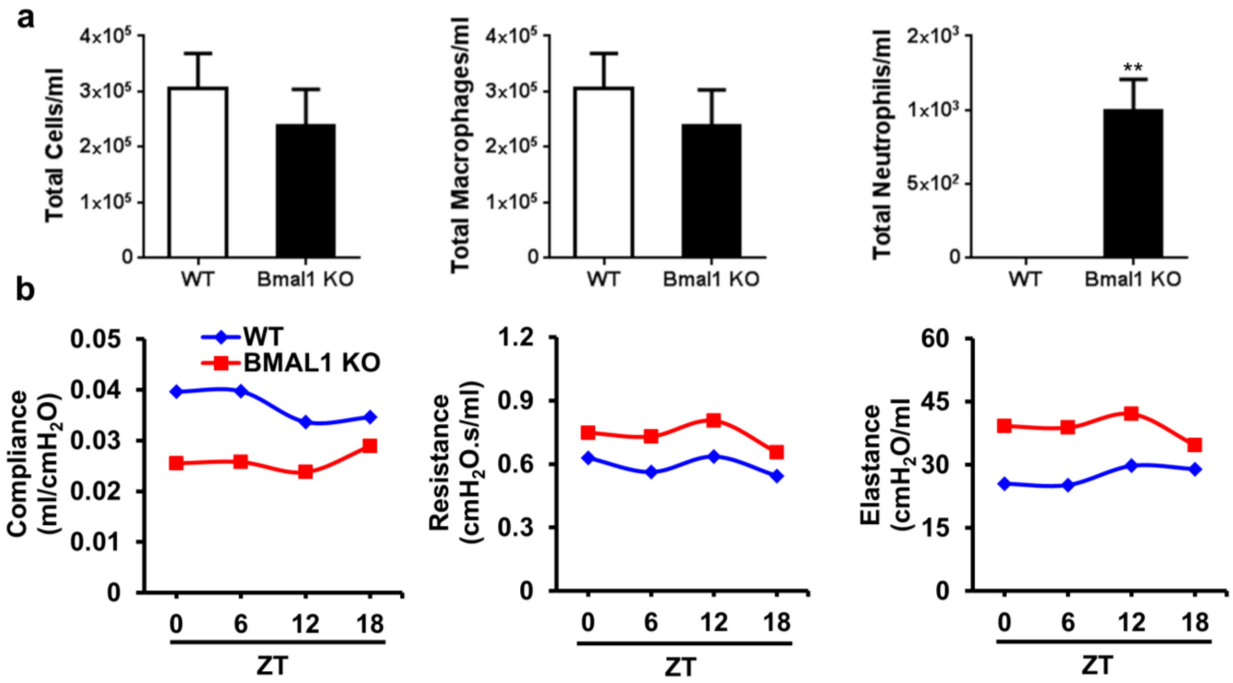
a
Total Cells/ml
4x10⁵
3x10⁵
2x10⁵
1x10⁵
0
WT
Bmal1 KO
Total Macrophages/ml
Total Neutrophils/ml
2x10³
1x10³
5x10²
**
b
WT
BMAL1 KO
Compliance (ml/cmH2O)
Resistance (cmH2O.s/ml)
Elastance (cmH2O/ml)
ZT

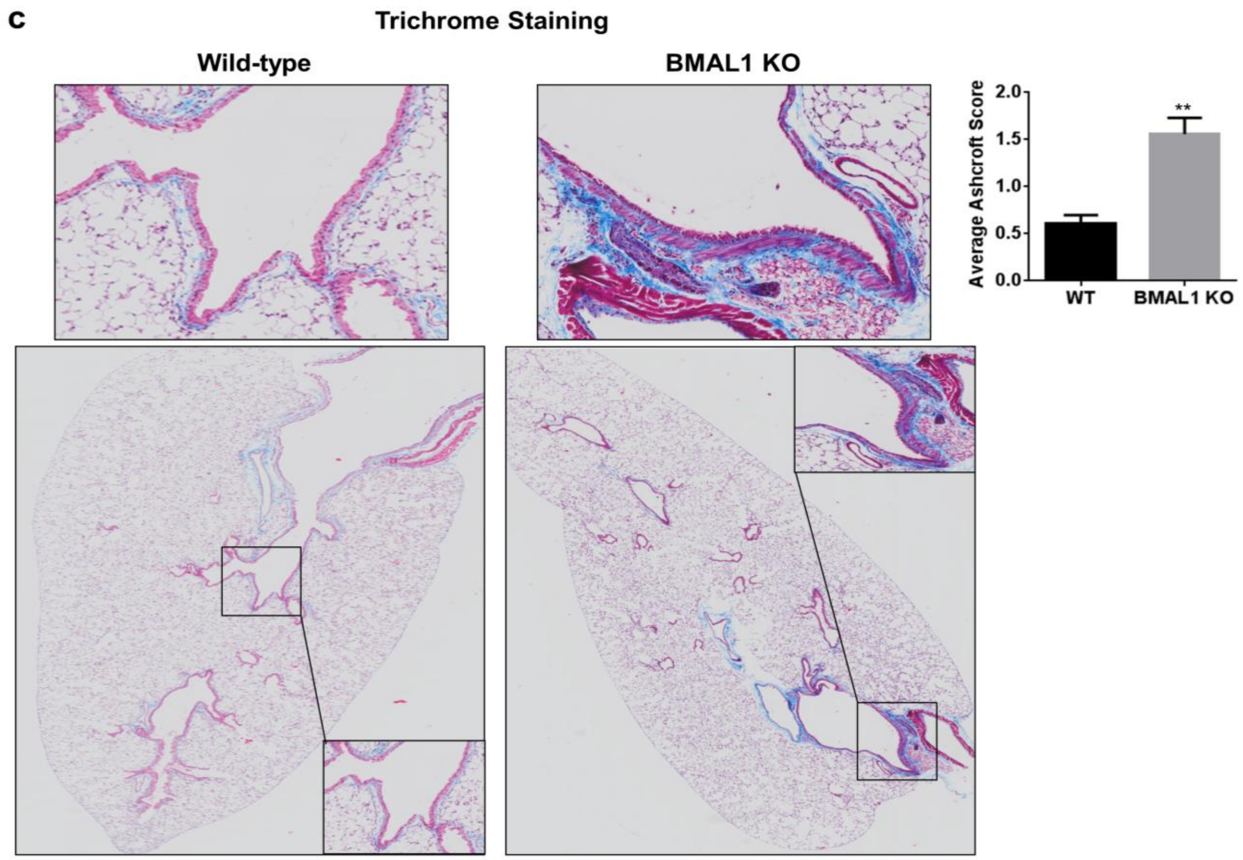

Supplementary Fig. 10. BMAL1 KO mice show increased neutrophil influx and a decline in lung function in conjunction with a pro-fibrotic lung phenotype. Data from WT littermates and BMAL1 KO mice (2-3 months old) euthanized at different time points (ZT0-ZT18) are shown. **(a)** The total cells in BAL fluid from WT and BMAL1 KO mice were averaged from all the ZT time points. At least 500 cells in the BAL fluid were counted on cytospin slides stained with Diff-Quik to determine the total cells, total macrophages, and total neutrophils. Data are representative of mean ± SEM (n=4/group). **(b)** Daily rhythms of lung compliance, resistance and elastance were measured in WT and BMAL1 KO mice at different ZTs (n=1/ZT0-ZT18). Both WT and BMAL1 KO mice displayed lung functions rhythms that peak at similar times across the day. **(c)** Trichrome staining in lungs of WT and BMAL1 KO mice. Representative WT and BMAL1 KO mouse whole lung sections (bronchial and alveolar parenchyma) stained with Gomori's Trichrome. A pro-fibrotic-like injury was observed in lungs of BMAL1 KO mice compared to WT littermates. Data are representative of mean ± SEM (n=4-5 mice/group). ** $P < 0.01$ significant compared to WT littermates.

Supplementary Tables

Supplementary Table 1. Lung compliance measured during different day and night ZT time in chronic air- and CS-exposed mice infected with influenza A virus

Day / Night ZT (h)	Compliance (mL/cmH_2O)			
	**Air** ‡	**CS** ‡	**Air + Virus**	**CS + Virus**
**ZT6 + ZT24**	0.051 ± 0.002	0.052 ± 0.002 ##	0.044 ± 0.003	0.045 ± 0.005
**ZT12 + ZT18**	0.046 ± 0.001	0.052 ± 0.002 $$	0.051 ± 0.002	0.039 ± 0.005 **, §

Data are shown as mean ± SEM (n=4-6 per group).

§ $P < 0.01$, significant compared to air (ZT6 + ZT24); ** $P < 0.01$, significant compared to air + virus (ZT12 + ZT18); ## $P < 0.01$, and $$ $P < 0.01$, significant compared to CS + virus (ZT12 + ZT18).

‡ Data from animals exposed to air/CS for 6 months from a previous study were included here for comparison [30].

Supplementary Table 2. Lung resistance measured during different day and night ZT time in chronic air- and CS-exposed mice infected with influenza A virus

Day / Night ZT (h)	Resistance ($cmH_2O.s/mL$)			
	**Air** ‡	**CS** ‡	**Air + Virus**	**CS + Virus**
**ZT6 + ZT24**	0.732 ± 0.009	0.697 ± 0.030 $^{\#\#\#}$	0.825 ± 0.014	0.723 ± 0.042 †
**ZT12 + ZT18**	0.795 ± 0.045	0.708 ± 0.038 $^{\$\$\$}$	0.703 ± 0.026	1.038 ± 0.154 $^{***, §, \$}$

Data are shown as mean ± SEM (n=4-6 per group).

§ $P < 0.05$, and § $P < 0.01$, significant compared to air (ZT12 + ZT18) and (ZT6 +ZT24) respectively; $^{\$}$ $P < 0.05$, and ***$P < 0.001$, significant compared to air + virus (ZT6 + ZT24) and (ZT12 + ZT18), respectively; $^{\#\#\#}$ $P < 0.001$, and $^{\$\$\$}$ $P < 0.01$, significant compared to CS + virus (ZT12 + ZT18); † $P < 0.01$, significant compared to CS + virus (ZT12 + ZT18).

‡ Data from animals exposed to air/CS for 6 months from a previous study were included here for comparison [30].

Supplementary Table 3. Tissue elastance measured during different day and night ZT time in chronic air- and CS-exposed mice infected with influenza A virus

Day / Night ZT (h)	Elastance (cmH_2O/mL)			
	Air ‡	CS ‡	Air + Virus	CS + Virus
**ZT6 + ZT24**	19.584 ± 0.724	19.201 ± 0.658 ##	23.391 ± 1.566	23.231 ± 2.556
**ZT12 + ZT18**	21.905 ± 0.359	19.502 ± 0.565 $$	19.808 ± 0.860	29.264 ± 5.089 ***, §

Data are shown as mean ± SEM (n=4-6 per group).

§ $P < 0.05$, § $P < 0.01$, significant compared to air (ZT12 + ZT18) and (ZT6 +ZT24); *** $P < 0.001$, significant compared to air + virus (ZT12 + ZT18); ## $P < 0.01$, and $$ $P < 0.01$, significant compared to CS + virus (ZT12 + ZT18).

‡ Data from animals exposed to air/CS for 6 months from a previous study were included here for comparison [30].
